# “At a Glance:” The Role of Diagrammatic Representations in Eugenics Appropriations of the “Infamous Juke Family”

**DOI:** 10.1007/s10739-023-09755-3

**Published:** 2024-02-12

**Authors:** Andrea Ceccon

**Affiliations:** Institute for the History of Medicine and Science Studies, University of Lübeck, Lübeck, Germany

**Keywords:** Eugenics, Jukes, Pedigrees, Arthur Howard Estabrook, Richard Louis Dugdale

## Abstract

The case of the Juke family is one of the most notable episodes of the history of eugenics in the USA. The Jukes were initially brought to the fore in the 1870s by a famous investigation that aimed at estimating the interplay of heredity and environment in determining the problems of poverty and crime. This inquiry triggered a harsh confrontation between two polar interpretations of the study, an “environmentalist” one and a “hereditarian” one. It was with the later reassessment of the case made by the Eugenics American Office (ERO) in the 1910s that the controversy was considered closed with the victory of the eugenicists’ hereditarian stance. As a result, the family was made a living proof of the alleged hereditary nature of crime and pauperism and a case study in support of the eugenicists’ plea for the sterilization of people deemed the bearers of hereditary defectiveness. In this article, I explore the role played by pedigrees and other diagrammatic representations in the eugenicists’ appropriation of the meaning of the case of the Juke family and the role played by this appropriation in asserting the superiority of the ERO’s method of work over rival approaches.

## The Most Infamous Family: Introduction

Exemplary case studies have always been high on the wish list of scientific and medical discipline formation and eugenics was no exception. Having well-rounded case studies available could, for example, play a significant role in the training of practitioners. However, since conducting breeding experiments with human beings, as it was done with plants or animals in the agricultural sciences, was ethically and practically impossible, other ways had to be found in order to obtain meaningful and effective textbook examples.^1^ One of these ways consisted in the collection of family pedigrees in order to trace inheritance patterns; therefore, establishing exemplary cases often took the form of charting pedigrees of so-called problem families in order to show how their “unit characters” were passed down from generation to generation.^2^

Given the sheer scale of work that this task could imply, its success depended on the crafting of educational programs for training a significant number of assistants that could help in the task. In the United States, the Eugenics Record Office (ERO)—one of the most influential centers for eugenics research in the world—established training summer programs for instructing college students as eugenics caseworkers since its founding in 1910.^3^ Here, one of the ways students were made acquainted with the eugenic mindset was by practicing on famous “defective stocks.” As a consequence, model “problem families” became both the training ground and the desired output of the courses. As an example, during the second training program in the summer 1911, participants were asked to provide an analytical study of one of America’s most well-known “dysgenic” families. They had to establish a numerical “index of conformity” of some of the family’s “special qualities” on the assumption that these were inherited in a Mendelian manner. They had to explore, on the basis of Bateson’s presence and absence theory,^4^ the inheritance of some of the family’s “unit characters” such as “temperament,” “remembering,” “domestic arts,” “literary capacity,” “calculating capacity,” “mechanical ability,” and similar features.^5^

**Fig. 1:**
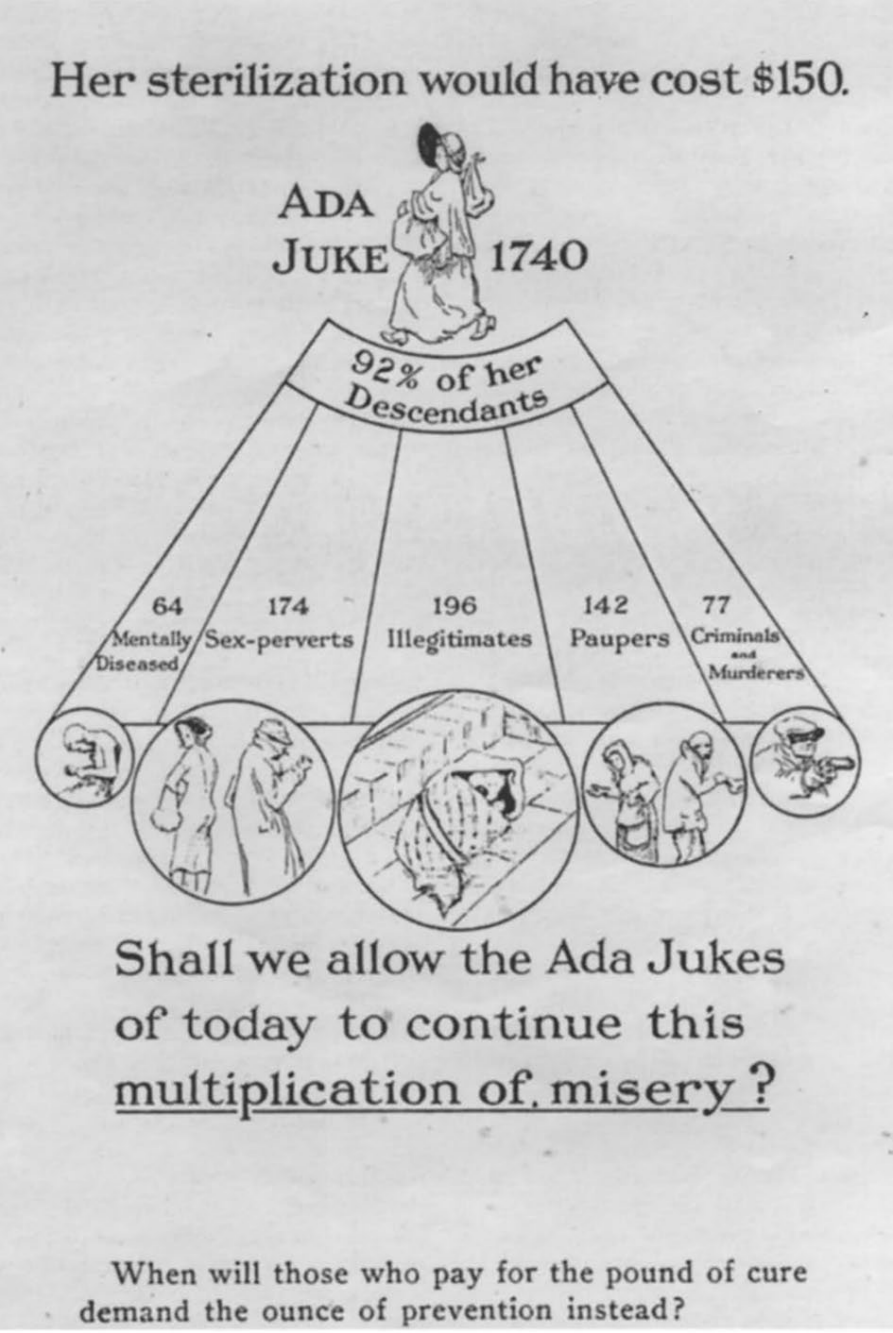
A widely reproduced poster portraying the legacy of Ada Juke (Norton 1937)

The family that was chosen for the exercise was one that was held to be a paragon of degeneration, vice and immorality. In Francis Galton’s words, it was the “extraordinary example” of a stock “apt to go to the bad” and of the “perpetuation of a criminal class by heredity” (Galton 1883, p. 63): the “infamous Jukes family” (Galton 1877, p. 347). Indeed, in the late 19th century and early 20th century, the Jukes became known as the embodiment of degeneracy. All of well-educated society knew what the Jukes meant or stood for. Specialist journals of medicine, law or education and popular magazines alike were eager to periodically revive the story and the meaning of this emblematic “stock.” Particular attention was devoted to a woman known under the pseudonym of Ada Juke, who was often designated as the downright origin of the famous dysgenic lineage (Fig. 1). As an article in the popular magazine *Scientific American* put it, “Ada Jukes [sic] [was] known to anthropologists as the ‘mother of criminals’” since it was deemed that one thousand two hundred persons directly descended from her, of which “one thousand were criminals, paupers, inebriates, insane, or on the streets” (Anonymous 1911, p. 562).

The reason why the Jukes were so renowned was that the family had been the object of the first study in a booming field of research providing eugenicists with many exemplars of degeneration: the “problem family” case studies (Rafter 1988). Published in 1877, *The Jukes: A Study in Crime, Pauperism, Disease and Heredity* is the work of Richard Louis Dugdale, a “merchant and manufacturer” with a keen desire “for the methodical study of social science.”^6^ However, despite the status of becoming a classic of eugenics, it is likely that Dugdale would have never called himself a “eugenicist,” and not simply because the term would be coined only a few months before he died.^7^ In fact, Dugdale declared that, in order to obtain a comprehensive knowledge for the prevention of crime and pauperism, it was necessary to evaluate the “entire facts embraced in the two main branches of inquiry into which the subject necessarily divides itself: The Heredity that fixes the organic characteristics of the individual, and The Environment which affects modifications in that heredity” (Dugdale 1877, p. 11). Indeed, as stressed by geneticist and historian Elof Axel Carlson (2001), Dugdale’s main point was to show that what was inherited was bad environment or the “tendency of heredity is to produce an environment which perpetuates that heredity” (Dugdale 1877, p. 64).

Because of this two-pronged approach to the debate that Galton formalized a few years before under the banner “Nature vs Nurture” (Galton 1874), the case was subjected to two polar interpretations after Dugdale’s death. The first one was given by those who, at the time were defined as “environmentalists” and it was based on the significance that Dugdale’s conclusions conferred to education, penitentiary reform and general public health reform. The opposite interpretation was given by advocates of eugenics, who claimed that Dugdale was on their side from the very moment the term eugenics was coined in Francis Galton’s *Inquiries into Human Faculty and Its Development* (Galton 1883). Here, Galton presented *The Jukes* as the example par excellence showing that crime and pauperism were a hereditary phenomenon. Indeed, in spite of the fact that Dugdale explicitly attacked the “extremists” who attributed all psychological and social traits to biological heredity (Dugdale 1876, p. 82), *The Jukes* was more and more read as a compelling proof of hereditary defectiveness. Soon, the environmental position seemed to have lost the battle for the meaning of the emblematic work. As a result, the family became one of the best-known case-studies meant to demonstrate so-called “hereditary crime” and “hereditary pauperism.” This understanding continued, in various degrees and with notable exceptions, at least until 1980, when Carlson wrote a short article stressing the “mystifications” and “distortions” to which the research had been subjected, to the point that he considered the text as the root of the hereditarian-environmentalist controversy in the United States (Carlson 1980). It was not until the beginning of the 21st century, when the names of the Juke family members were found in the “Estabrook’s papers” in the archives at the State University of New York,^8^ that it could finally be shown what was actually known since the beginning, namely that the Jukes were not even a family (Christianson 2003; Dugdale 1877, p. 14; Jarvenpa 2018, p. 20).

However, even in the late 19th and early 20th century not everybody was convinced that Dugdale’s text proved the strict biological character of social problems. For example, in his influential textbook *Genetics and Eugenics*, the geneticist William Ernest Castle stated that the Jukes’ case, though often cited to show the persistence of the traits of one individual through several generations, was more chiefly due to environmental factors and intermarriage within the family environment (Castle 1916, p. 269). Indeed, some supporters of eugenics were well aware that, as the famous eugenicist and psychologist Henry Herbert Goddard put it, “so far as the Jukes family [sic] is concerned, there is nothing that proves the hereditary character of any of the crime, pauperism, or prostitution that was found” (Goddard 1912a, p. 53). Many social reformers too, were well aware of this lack of evidence, and they did not lose hope of pushing an alternative, environmentalist reading of the case. Indeed, as the educational and social reformer Mary Carpenter stressed, the very originator of the epithet “mother of criminals”—Dugdale’s mentor Elisha Harris—was himself an environmentalist. In fact, Harris firmly maintained:

[D]ebased as the [Juke] stock became in the successive generations, I have reason to believe, that, in any of the generations, most of the individual members in it could have been rescued and saved from vice and offences by a prompt and reasonable care and training of the children. (Carpenter 1875, p. 79)

Moreover, the sociologist Franklin Henry Giddings, who wrote the introduction to the 4th edition of Dugdale’s book (the one published the same year when the Eugenics Record Office was founded) warned readers that “an impression quite generally prevails that ‘The Jukes,’ is a through-going demonstration of ‘hereditary criminality,’ ‘hereditary pauperism,’ ‘hereditary degeneracy,’ and so on. It is nothing of the kind, and its author never made such claim [sic] for it” (Dugdale 1910, pp. iii–iv). Giddings was referring to “eugenicists such as Oscar McCulloch, David [Starr] Jordan, and Charles [Benedict] Davenport who had claimed Dugdale as one of their own” (Ryan 1999, p. 25).

In light of these controversies, it is not difficult to understand why Davenport, who was the influential founder and director of the ERO and arguably one of the most prominent eugenicists of the world, deemed that bringing Dugdale’s study up-to-date under the aegis of his institution would have been of great benefit to the cause of American eugenics. Through representatives of the Carnegie Institute, of which the ERO was part, Davenport managed to obtain Dugdale’s original manuscript containing the real names of the Juke members which that document alone could provide, Jukes being a pseudonym invented by Dugdale to protect the identity of the people involved.^9^ Davenport assigned to Arthur Howard Estabrook, one of his field workers freshly trained in the first ERO summer program,^10^ the ambitious task of updating *The Jukes* and revising its conclusions in a way so compelling that no opponent would ever be able to question the hereditarian significance of the emblematic case again.

The product resulting from this project was Estabrook’s *The Jukes in 1915*, which was presented as a follow-up investigation of Dugdale’s work (Estabrook 1916). Estabrook claimed that the book simply updated the research with the study of the last generations of the family. One of the most important arguments advanced to justify the redoing of the inquiry was that, whereas until Dugdale’s time the Jukes had always lived in a small, isolated valley with a “backward” society, the last two generations spread all over the United States, thus enabling Estabrook to really compare the relative importance of environment and heredity. Estabrook, however, was very careful in not making explicit claims about overturning Dugdale’s conclusions and the text was, on the contrary, described as a neutral and objective presentation of the facts of the lives of the Jukes.

Among these facts, a key role was played by what Harry Hamilton Laughlin, who was first the superintendent and then the assistant director of the ERO for the entire lifespan of the institution, would later define as “pedigree-facts” (Laughlin 1922). In contrast to Dugdale, who chose to visually display his large amount of data in the form of tabular family charts, Estabrook introduced in the Jukes’ tale the modern pedigree-study as it was advocated at that time by the Eugenics Record Office. In so doing, Estabrook could make use of the powerful imagery techniques that were being developed at the ERO and capitalize on what Veronika Lipphardt has called the “deterministic potential” of these kinds of visualizations (Lipphardt 2015, p. 49), making an effective method of persuasion of them. In fact, genetic pedigrees were often presented as straightforward evidence that like engendered like. As an educator and first Principal of the UCL Institute of Education John Adams wrote in 1910,

the straight line has a special illustrative value. In dealing with mental activity we find that sense of direction is characteristic of mental functioning [and] it may fairly be said that the straight line in certain diagrams performs the functions of those signs of direction. In a genealogical table the lines really do direct the mind, which in following this direction shows itself to be in this case passive. (Adams 1910, p. 386)

The following sections take a closer look at the ERO’s reappraisal of Dugdale’s first investigation through the lens of the radical shift in the diagrammatic representations that Estabrook conducted. Besides being an effective way of communicating research outcomes and a very useful method for mastering a large amount of information, diagrams can also be used to suggest a mechanistic inference framework. In the case in point, as we will see, they were also used as a way to promote the exclusive authority of one preeminent method of work over other possible approaches to social problems—as well as over other possible approaches to eugenics itself—and to promote the institution associated with that method over rival actors. In the next section, I will briefly examine Dugdale’s original inquiry and in the following one I will describe the entry of the Jukes in the ERO’s pedigree method. Subsequently, after having examined an early attempt made by Davenport of bringing the famous case to the side of the eugenicists, having briefly explored how the ERO dealt with problems of credibility and having briefly examined questions of pedigree standardization and the creation of a visual identity, I finally investigate how the reinterpreted Jukes case and the use of diagrams could be instrumental in asserting institutional power.

## Charting the Rivers of Nature and Nurture: Dugdale’s Jukes in 1877

As already mentioned, Dugdale started to work on the project under the supervision of Elisha Harris. Harris, a physician, statistician and public health reformer, was one of the originators and the first secretary of the American Public Health Association (Anonymous 1884). It was however as secretary of the Prison Association of New York that in 1871 he launched an investigation on the “relationships of pauperism and vice to crime and disorder.”^11^ During this enquiry he thought he could highlight patterns of descent in the records of jails and poorhouses. He could trace some inmates of different prisons in the Hudson Valley as descendants of Margaret, a young woman rejected as she sought “government assistance in the face of impeding childbirth” (Lombardo 2012, p. 210). Legal historian Paul A. Lombardo argued that Margaret’s story was a way for Harris to sound the theme of prevention. Showing to the public the plight of a destitute girl who was abandoned by the public authority, Harris warned that this was an illustration of the origin of a “race of criminals, paupers and harlots,” and the tale meant to show what could be done to prevent it.^12^ He therefore claimed that the likes of Margaret should be saved from corrupting environment and from the life of poverty and crime that those living conditions entailed for her and for her progeny. The story had a certain visibility and could find its way in venues such as the *New York Times.*^13^

The following year Harris passed the investigation to Dugdale, who in the meanwhile joined the Prison Association as a member of the executive committee.^14^ As Lombardo stressed, Dugdale was a social reforms enthusiast whose goal was to amass “sufficient fortune to purchase the privilege of independent subsequent inquiry.”^15^ In one of the Hudson Valley prisons, Dugdale found six people, under four different family names, that turned out to be related. He traced these prisoners’ family roots and established that they reached back to the early colonists, determining a long lineage that had little intermarried with the rest of the population and had never moved from the “ancestral breeding spot” (Dugdale 1877, p. 13). Dugdale could trace the “origin of the stock of the ‘Jukes’” to a man called Max, a descendant of early Dutch settlers (Dugdale 1877, p. 4). Two of Max’s sons married two out of six sisters; five of these sisters (called with the fictitious names Ada, Bell, Clara, Delia and Effie—the sixth could not be identified) are those to which the pseudonym Juke was given and whose progeny are what Dugdale called “the Jukes blood.” He identified Margaret as the first of these sisters, his Ada. The author could find 709 members of the family and determine “distinctively industrious, distinctively criminal, distinctively pauper, and specifically diseased” lines (Dugdale 1877, p. 16). He deemed that the descendants of one of Ada’s illegitimate children were the starting point of the distinct criminal strand of the degenerate family and he estimated the cost that the society had to bear for the needs and the crimes of the family, an aspect of the study that particularly attracted public interest.^16^

**Fig. 2:**
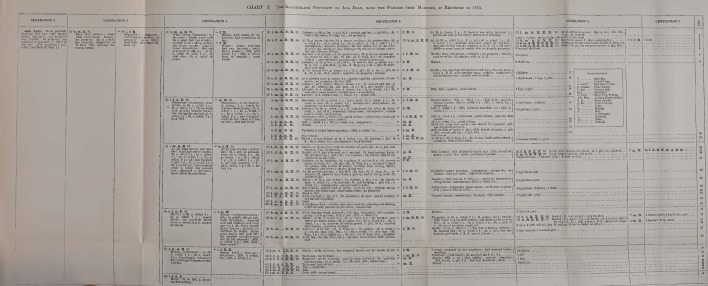
Ada Juke’s illegitimate legacy (Dugdale 1877, between pp. 14 and 15)

**Fig. 3:**
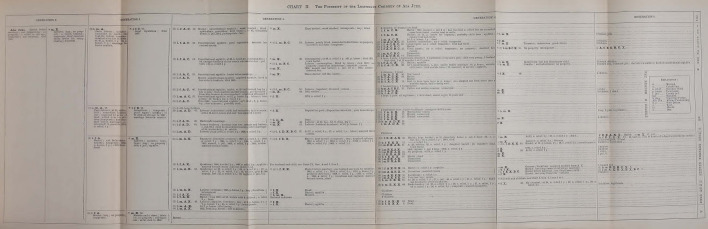
Ada Juke’s legitimate legacy (Dugdale 1877, between pp. 16 and 17)

One of the ways Dugdale tried to convey the meaning of his work was through the visual strategy with which he synthesized his findings. The visual apparatus of his text was not conspicuous; indeed, *The Jukes* showed only four family charts in tabular form. However, the author estimated that they would be the tool allowing him to show synthetically how the events in the life of parents are reproduced in the “career” of their children (Dugdale 1877, p. 24). In Dugdale’s words, the four charts had to be considered the keystone “upon which the entire present study is based” (Dugdale 1876, p. 83). The main function he assigned them was to make strict comparisons between “the life of [an individual] and that of his generation or his posterity so that any characteristic which is hereditary will thus be revealed” (Dugdale 1877, p. 12). Accordingly, the charts were structured in a way enabling the visual depiction of “comparable facts” (Figs. 2 and 3).

In Dugdale’s intention, the simple tabular arrangement should have made possible to compare “generation to generation, condition to condition, sequence with sequence” (Dugdale 1877, p. 16), thereby bringing into prominence on the one hand the features occurring continuously that might be attributed to heredity, and, on the other hand, the effects of environment. The family charts thus conceived were deemed to be “the surest way to exclude preconceptions” because they could exclude “artificial arrangement, accepting what came as it came” (Dugdale 1876, p. 83).

The main axis of organization of the charts is the division into columns, which have to be read from left to right. Each main column shows the various generations of the lineage, which in turn are further divided in two columns, the first one showing the direct descendant of the preceding generation and the second one showing their partners (but not the direct ancestors of these partners, who are excluded from the analysis). Each of the four charts represents one specific “stock” or “line” of the Juke family. The first one depicts the lineage of Ada’s illegitimate son who, as mentioned, was defined as “the progenitor of the distinctive criminal line” (Dugdale 1877, p. 15). The chart however, despite being identified as the one concerning Ada’s son’s descendants, does not start the lineage with him, but with Ada herself, without any consideration for the unknown father. Representing Ada as the only member of that generation, the chart visually depicts the stock as budding off directly from her. Given that the project began specifically as Harris’ investigation on “the mother of criminals,” speculations about the primacy of her role are not difficult to raise. Furthermore, Ada is described as the second generation, even though no mention is ever done of her possible parents.

The second chart represents Ada’s legitimate descendants and it starts with “Generation 2,” as well. Chart 3 considers Bell’s line of descent and chart 4 is about Delia’s lineage. Delia was one of the two sisters who married one of Max’s sons and therefore, in this case, the chart starts with Max himself, who is identified as “Generation 1.” It has to be noted that Dugdale never described Max as the “progenitor” or the “common ancestor” to whom the Juke family can be traced. In fact, being the father of the husbands of two Juke sisters’ made him everything but a common ancestor, especially if we consider that one of the lines that were deemed more “interesting” was that descending from Ada’s illegitimate son, with whom Max had no direct relation (except the intermarriages between his sons’ line and that of Ada’s son). Nonetheless, the way the charts are built clearly makes him the family’s first generation, which indeed was how later readers of the book considered him.

Beside the members I have already named, the other individuals plotted in the charts are identified and characterized by a notational system, a fundamental aspect of which is the fact that it was devised to “discriminate the stocks.” Dugdale drew a distinction between the “Jukes blood” (the direct descendants of the five sisters), identified by the first five letters of the alphabet in block letters (A for Ada, B for Bell, C for Clara, D for Delia and E for Effie) and the “X blood,” which is composed of the family members outside the Jukes line, but who married a Juke (and are identified with the letter X). This distinction is particularly important because it allows the reader to consider the intermarriages between the five lines and it has been often referred to as one of the main reasons why the work could be interpreted as the proof of a rigid hereditarian transmission of social and psychological characters. Using this system, the offspring resulting from any given mating contains all the letters representing their ancestral derivation. Each individual is then identified with numbers according to the order of his or her birth and is characterized by a letter indicating whether he or she is “bastard” or “legitimate.” Legitimate unions are symbolized with “×” and illegitimate ones are symbolized with “=.” So, for example, (4) l.f.A.C.B.C. × (7) b.m.B.C.X. is a legitimate union between a fourth child, a legitimate female descending from the Ada’s, Bell’s and Clara’s stock (with both her mother and father having Clara as grandmother—that is, the double presence of the letter C immediately enables the reader to identify a cousin marriage) and a seventh child, an illegitimate male descending from Bell, Clara and having one of the two parents whose ancestors are not part of the Jukes.

The charts so conceived become a tool for mapping consanguinity, endogamy and crossing. Dugdale deemed this visual arrangement particularly fitted to find characteristics that are peculiar to specific lines. In fact, the author was persuaded that it was possible to find lines of descent that are prone to criminality, others that are distinctly pauperized and still others that are particularly industrious. Following the charts from the first generations to the left to the contemporary ones to the right, Dugdale aimed at charting these features as they flow down the rivers of descent, “the breaks in the line at certain points indicating with great precision the modifying effects of disease, training, or fortuitous circumstance which have intervened or changed the current of the career” (Dugdale 1877, p. 16).

It has to be noticed that, as Dugdale clearly states in the preface of the book, the Juke family is actually the aggregation of “forty-two family names included in the lineage to one generic application” for the convenience of treatment (Dugdale 1877, p. 2). We have also to remark that the family was defined as the descendants of six sisters “whose parentage has not been absolutely ascertained” (Dugdale 1877, p. 14). In fact, it seemed that they were not all full sisters and that some of them (at least two) were illegitimate. Concerning the legitimate ones, their relation was established by the means of their surname, which may mean that they could have been half-sisters as well on the mother’s side, or even that they could have not been sisters at all. In addition, the sixth sister, having left the country, was not considered part of the family because she turned out to be impossible to trace—and she would have been an external element compared to the very local character of the family.

## The Wheel of Vice, Crime and Pauperism: Estabrook’s Jukes in 1915

**Fig. 4:**
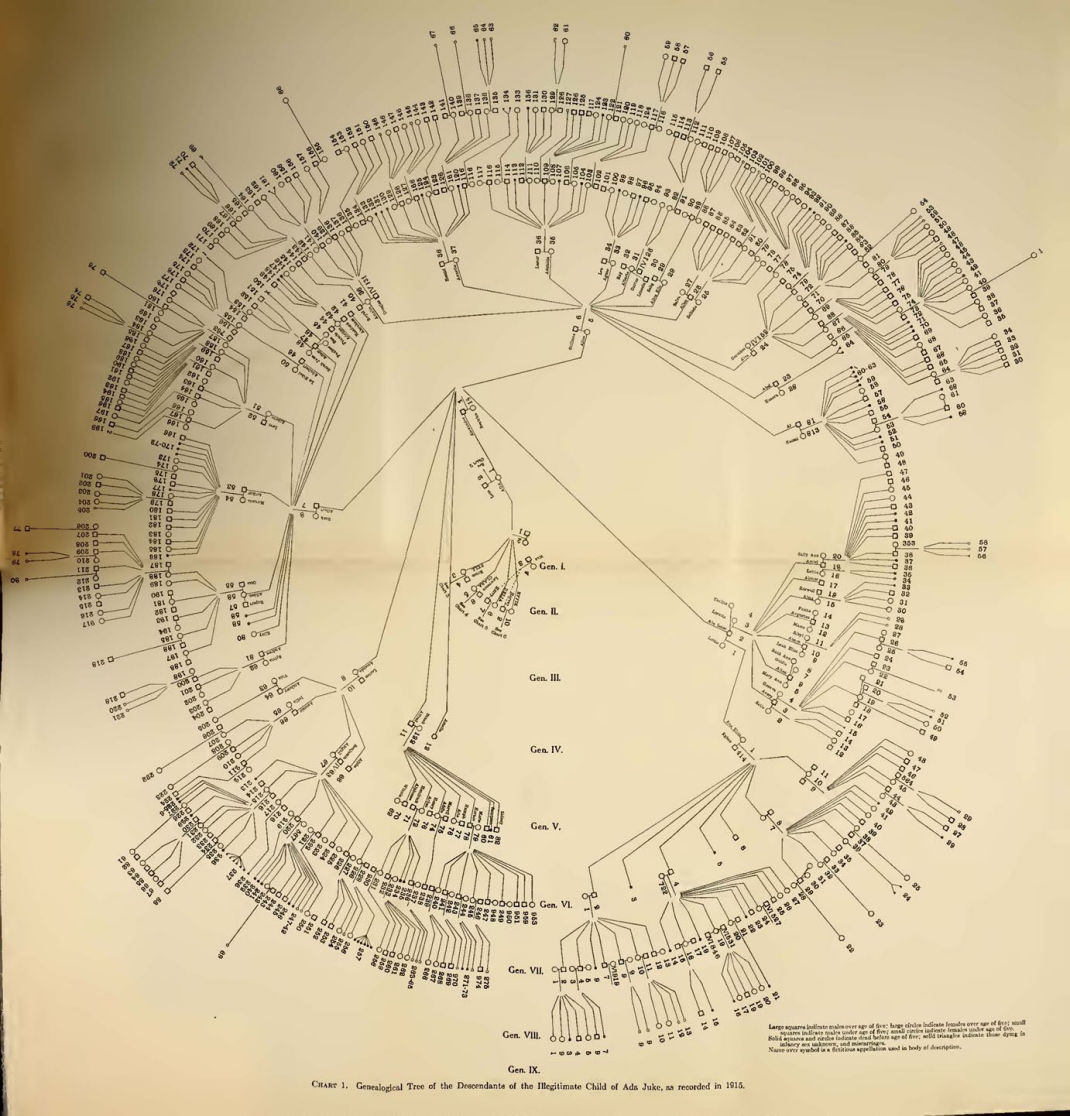
Genealogical tree of the illegitimate descendants of Ada Juke (Estabrook 1916, between pp. 4 and 5)

**Fig. 5:**
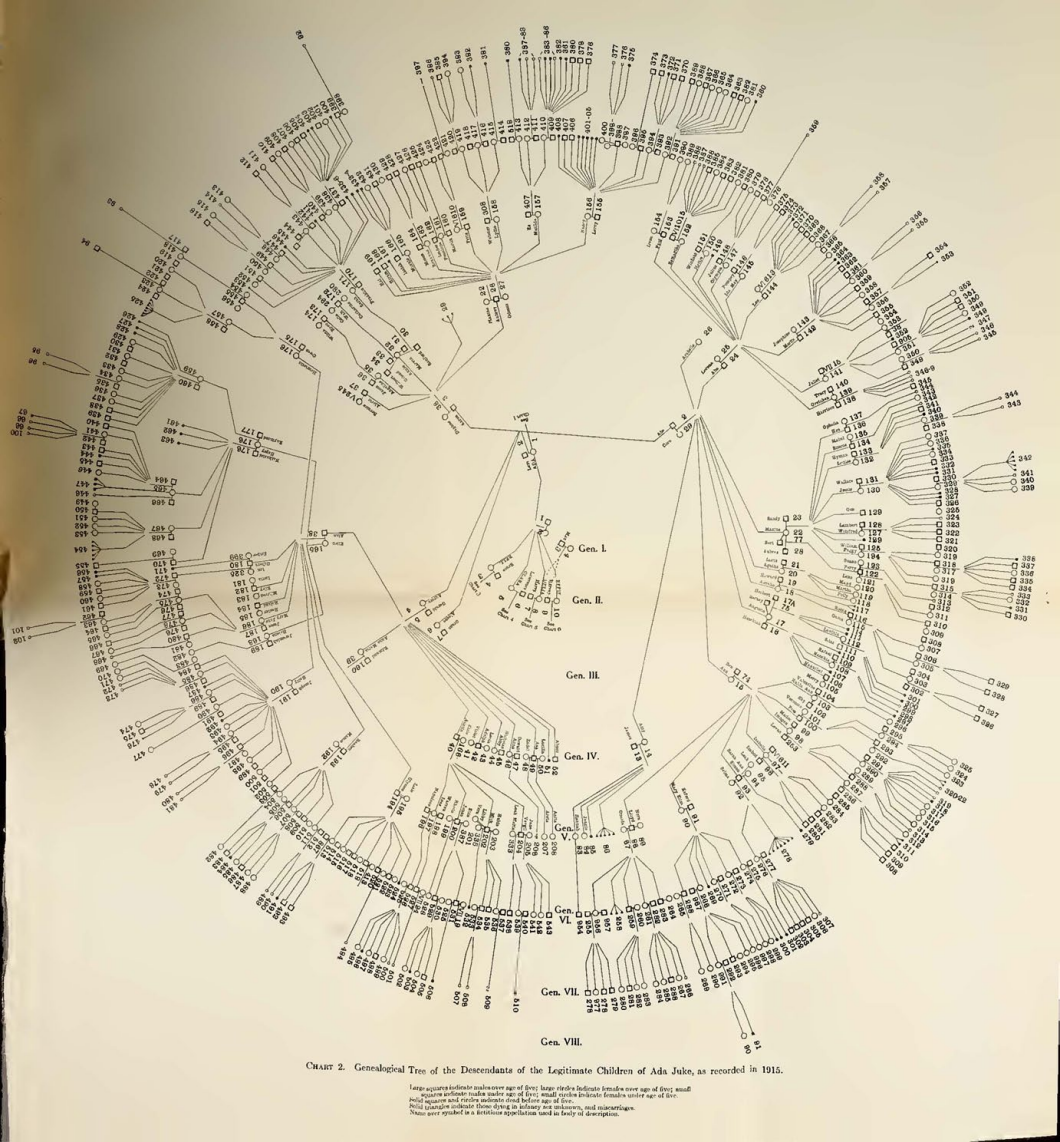
Genealogical tree of the legitimate descendants of Ada Juke (Estabrook 1916, between pp. 18 and 19).

Dugdale’s charts disappeared in Estabrook’s follow-up text. Here, the reversal of Dugdale’s conclusions about the need of educational, penal and public health reforms was matched with a radical restyling of the visual apparatus of his magnum opus. Estabrook chose to expose his version of the case by making use of two different sets of iconic diagrammatic tools: the new and picturesque “wheel charts” (Figs. 4 and 5), and the by then classical genetic pedigrees. The former sort had been already used in 1912 in Davenport and Harry F. Danielson’s *The Hill Folk; Report on a Rural Community of Hereditary Defectives* (Danielson and Davenport 1912) and, immediately later, in *The Nam Family. A Study in Cacogenics*, a text that Estabrook himself co-authored with Davenport (Estabrook and Davenport 1912). The use of these large and peculiar fold-out diagrams is explained with the necessity of pulling together the large number of people involved in the study in a way that allows the eye to grasp the whole situation “at a glance.” Furthermore, the geometrical arrangement and the almost aesthetic quality of their graphic approach of conveying the data cannot but have an inevitable impact on the reader. The anthropologist Robert Jarvenpa described the Nam wheel charts as resembling “a complex bicycle wheel with jagged spokes, an elaborate pinwheel, or a fantastic micro-organism with innumerable delicate spines or tendrils” (Jarvenpa 2018, p. 98).

At the center of all the wheel charts there is a core composed by the first two generations. Generation I is composed by Max, his unknown wife and the unknown couple which supposedly gave birth to the five Juke sisters. The latter define the first circle of the wheel and are connected to the “original couples,” as Davenport identified the two central pairs, by lines which diverge from the center. The descendants of the five Juke sisters are situated on the second circle, and the lines indicating their descendants diverge from the line uniting their parents. From the second circle outwards, the wheel diagrams differ from each other. In fact, even though the first two generations are always displayed as the common source that has always to be visually at hand, each diagram shows the stock deriving from one of the sisters. Their circular development from the center to the external circles is meant to suggest the expansion of the family. From the core of the Juke territory, its members scattered throughout the country, which was represented by the outwards movement of the wheel, with the local and “original” nucleus of the family at the center that progressively spread, through its offspring, all over the spaces around (Estabrook pointed out that he “examined” members of the family as far as New Jersey, Connecticut or Minnesota). The generations are numbered with Roman numerals, with the “common ancestors” as Generation I, while the individuals in each generation are enumerated with Arabic numerals. This second enumeration is independent for each generation, thus enabling the reference to individuals in the text by the generation number and their consecutive number in that generation. In cases in which an individual appears twice on the chart because of cousin marriages, he or she can always be designated by the number which indicates his descent.

The result is a complex diagram that is much less readable and less apt to detailed analysis than Dugdale’s charts; however, its synoptic value is undeniable and its intricacy and linear ramification doubtlessly conveys a sense of sophistication and rational organization of the data that Dugdale’s tabular charts could not match. By all means, a wheel chart “bespeaks virtuosity and scientific rigor” (Jarvenpa 2018, p. 98). In this respect, *The Jukes in 1915* was part of a project, directly coordinated by Davenport and intersecting with Goddard’s *The Kallikak Family*—arguably the only other family case study that acquired the same iconic status as the Jukes and that was published a few years before Estabrook’s book (Goddard 1912a). This enterprise aimed at creating a database of outstanding exemplary cases. The first of these was *The Hill Folks*, “the first of a projected series which is intended to embody some of the more extended researches of the Eugenics Record Office, especially such as, on account of extensive pedigree charts, require a page of large size” (Danielson and Davenport 1912, p. v). Within this framework, the Jukes played a pivotal role because of their huge emblematic value*.* The significance of revising Dugdale’s work was considered having stakes so high as to induce Davenport to warn Estabrook: “Let me urge the desirability of being more discrete [sic] than usual on this assignment. The notoriety of the family is great [...] Be wise as a serpent and harmless as a dove.”^17^

**Fig. 6:**
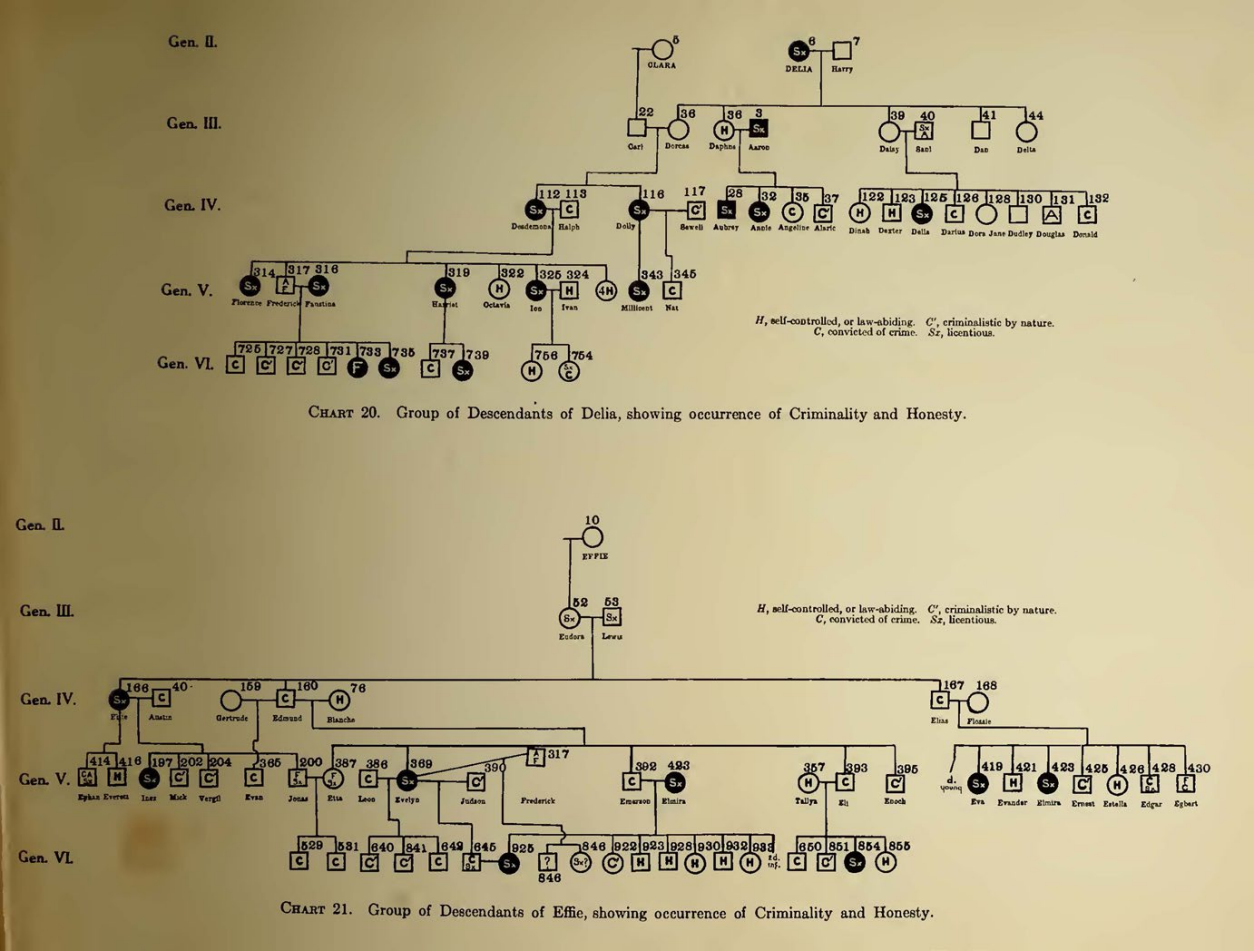
Occurrence of criminality and honesty in Delia’s and Effie’s descendants (Estabrook 1916, between pp. 66 and 67)

Besides the evocative wheel charts, Estabrook’s work shows also more common genetic pedigrees: 16 “small pedigrees” (as they were defined by Laughlin: diagrams that could be printed on the page together with the text; Fig. 6) and four large ones (requiring a separate folded-out sheet). These were the kind of pedigrees that have been defined by historian of medicine Pauline Mazumdar as tools for both investigation and propaganda in eugenics (Mazumdar 1992, p. 3). In particular, “small pedigrees” are the ones that practitioners of eugenics needed to produce and master. They were the actual “pedigree-facts” that could be realistically proposed to political bodies because they required embarking on less time-consuming and expensive researches. As already mentioned, “pedigree-facts” is a definition given by Laughlin, who was one of the most dynamic players in pushing an American eugenics policy agenda. As he revealed to the Committee on Immigration and Naturalization at the US House of Representatives on the occasion of the hearings for the Immigration Act of 1924, a eugenic study conducted by the means of a “short pedigree” had usually to involve 15 or 20 of prepositus’ near kin.^18^ That was what he deemed enough to throw some light upon the proband’s character in order to determine whether he or she would make a desirable addition to the population of the United States (Laughlin 1924, p. 1268).

**Fig. 7:**
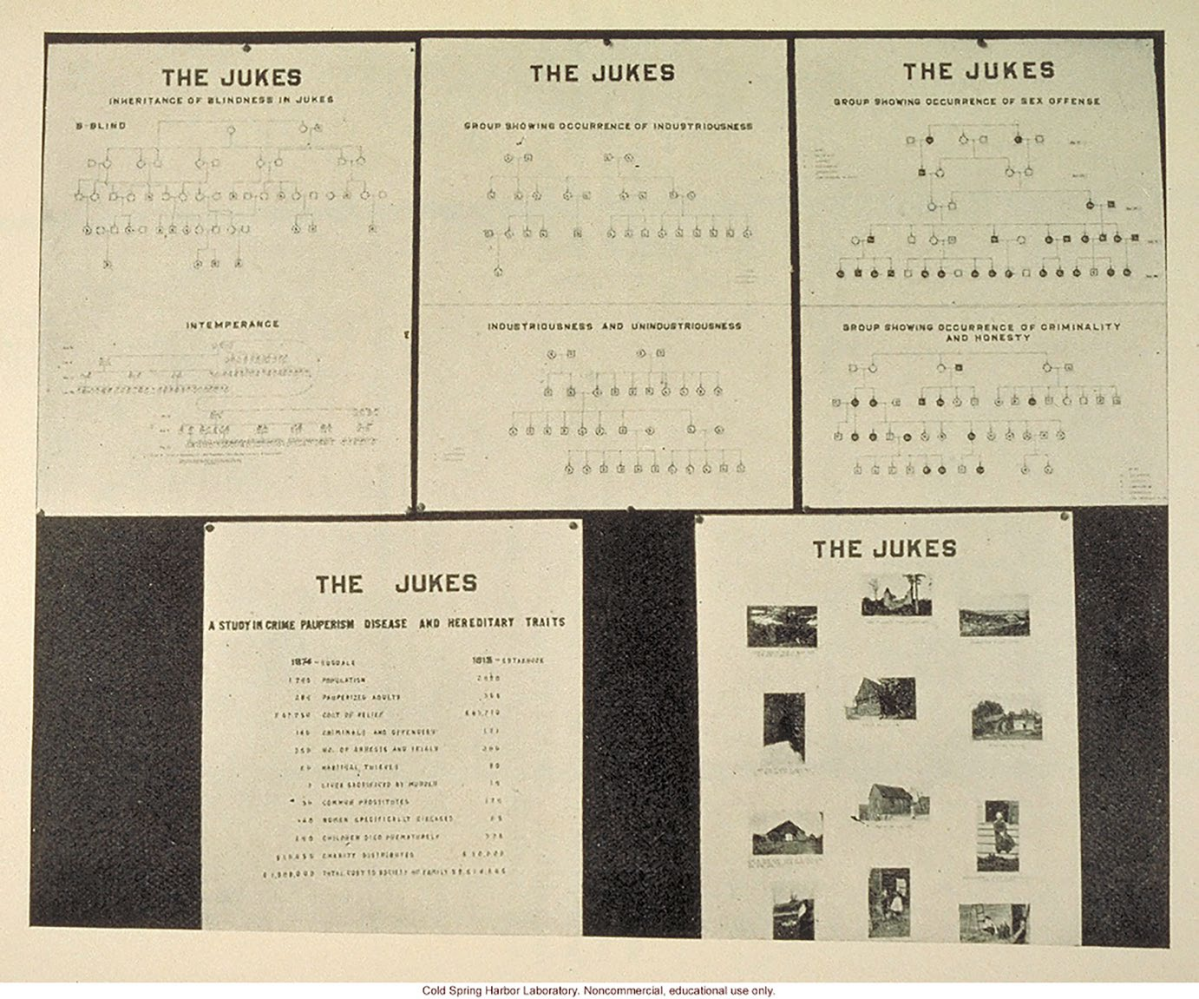
“The Juke charts compare the family as known to Dugdale in 1875 and again to A. H. Estabrook in 1915, forty years later ... Pictures of various members of the family and their living conditions are shown. By A. H. Estabrook” (Laughlin 1923, p. 148 (caption) and p. 149 (image))

The extent to which the ERO relied on the persuasive and demonstrative power of its pedigree-method can be seen from the use of the Jukes “small pedigrees” in the Second International Exhibition of Eugenics. Laughlin, as one of the most prominent finance committee members, played a major role in organizing the Exhibition. He aimed at assembling exhibits “relatively few in number but outstanding and specifically illustrating definite principles” and one of these exhibits “striking in nature” was assigned to Estabrook (Laughlin 1923, pp. 14 and 16). Obviously enough, it had to be on the Jukes (Fig. 7).

**Fig. 8:**
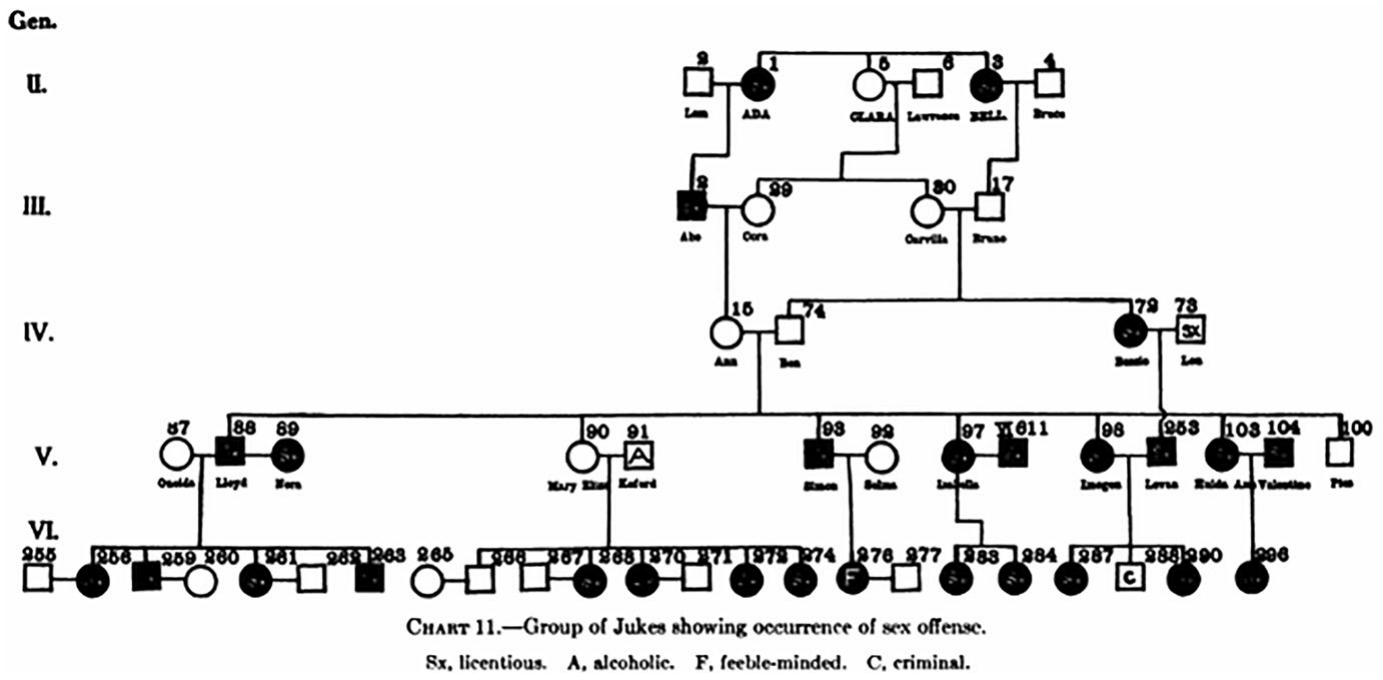
Pedigree showing the occurrence of sex offence (Estabrook 1916, p. 56)

The exhibit showed “short pedigrees” taken from *The Jukes in 1915* demonstrating the inheritance of physical characters such as blindness as well as “mental and moral faculties” such as intemperance, industriousness, unindustriousness occurrence of sex offence, criminality and honesty, etc. (Fig. 8).

These pedigrees were here used to compare the family as it was known to Estabrook to the family as it was known to Dugdale, whose findings were given in terms of simple numerical data and seemed dwarfed by the seemingly more sophisticated ERO “analytical tools.” Furthermore, the meaning of the “short pedigrees” was self-evident enough to be comprehended and appreciated by “the man of ordinary intelligence and education, but without special scientific training” all the while withstanding the “test of professional scrutiny” (Laughlin 1923, p. 16).

After all, as stressed by the historian of science and medicine Philip Wilson, Laughlin advocated the importance of using simple but rigorous and effective ways of publicly displaying knowledge since he was a college student (Wilson 2008, p. 167n). In an article published when he was only 19 for the local *The School Journal*, Laughlin encouraged younger students to foster their achievements “in a manner that will show each pupil at his best, but also in a manner that will show the progress made” (Laughlin 1899, p. 489). For that purpose, nothing was better than a “visible proof of progress” that could convey, in an iconic manner, the sense of increased knowledge acquired thanks to their work (Laughlin 1899, p. 489). In order to enhance the value of the pupil, research must be done from the outset with a view to effective communication. Therefore, if the public is to be interested, “that human trait which demands system must not be lost from sight;” the work, however, has also to be “displayed in a manner pleasing to the public” (Laughlin 1899, p. 489). Furthermore, “all work should be arranged for display in the most compact and accessible manner possible. Much work, such as historical panels and the like, while it must be able to stand inspection, must also be so displayed as to be comprehended at a glance” (Laughlin 1899, p. 489). In this regard, it seems that Estabrook was a model student, a claim that has to be taken at face value considering that he was trained as a eugenicist during the intensive summer programs organized by the ERO in which Laughlin, among many others, was teaching.

One of the major results that Estabrook could propose was the introduction in his book of “feeble-mindedness” as one of the most prominent of the Jukes’ hereditary features and its correlation with criminality. More to the point, Estabrook claimed that criminality was not a hereditary character in itself, but a consequence of the inheritance of feeble-mindedness. As the criminologist Nicole Hahn Rafter stressed, mental retardation was not one of the main focuses of Dugdale’s inquiry. In the original *Jukes*, “idiocy” and “imbecility” had not had a major impact on Dugdale’s considerations (Rafter 1988, p. 10). Indeed, as opposed to the eugenicists, Dugdale associated criminality not with mental retardation, but with excessive physical vigor (Dugdale 1877, p. 49). Furthermore, the loosely defined concept of feeble-mindedness such as it was generally used in eugenics became a more compelling framework of analysis only after the development and application of the Binet-Simon method of intelligence testing and the studies conducted by Goddard (see for example Goddard 1912b).

In fact, Estabrook’s argument budded from a remark on the Jukes case made by Goddard in *The Kallikak Family* (1912a). Goddard was a prominent eugenicist and psychologist, well-known for having established the term “moron” for clinical use and for having been one of the staunchest defenders of the hereditary nature of feeble-mindedness. In *Kallikak*, he regretted the fact that the Jukes had always lived in the same place because that made it impossible to establish what might have become of them if they had good training and environment. In his eyes, it was clear that the “question of the hereditary character of crime received no solution from the Jukes family” (Goddard 1912a, pp. 53–54). For his part, somewhat surprisingly, he claimed to believe that “criminals are made and not born” (Goddard 1912a, p. 86), and that what makes them is inherited feeble-mindedness. In other words, criminality itself was not an inherited character. Goddard believed that what was inherited was the tendency to reproduce the faulty environment responsible for the emergence of criminality and that this tendency is the result of inherited feeble-mindedness. Unfortunately, Goddard’s theory was in contrast with Dugdale’s assertion that the Juke family members had to be generally deemed “of normal mentality” (Goddard 1912a, pp. 52–53).

As a result, Goddard was persuaded that, granted the criminal character of many Jukes, Dugdale’s judgment concerning their “normal mentality” had to be wrong and the ERO was hoping that Estabrook’s research could provide the evidence that Goddard’s insight was right. This in turn could furnish a further demonstration of heredity and environment as integrated entities because the feeble-minded Jukes, which Estabrook esteemed to be half of the family, were “brought up under faulty environmental conditions which they consider normal, satisfied with the fulfilment of natural passions and desires” (Estabrook 1916, p. 85). The other half of the family, on the other hand, “perhaps normal mentally and emotionally, has become socially adequate or inadequate, depending on the chance of the individual reaching or failing to reach an environment which would mold and stimulate his inherited social traits” (Estabrook 1916, p. 85). Estabrook grounded his conclusions on the consideration that

if criminality is a recessive trait, a C X C mating [C stands for “being convicted of crime”] should produce 100 per cent criminal offspring. If self-control is recessive, then an H X H mating [H stands for “self-controlled or law-abiding citizens”] should give 100 per cent honest offspring [and] neither the C X C nor H X H matings give 100 per cent of that trait in the offspring. (Estabrook 1916, p. 63)

**Fig. 9:**
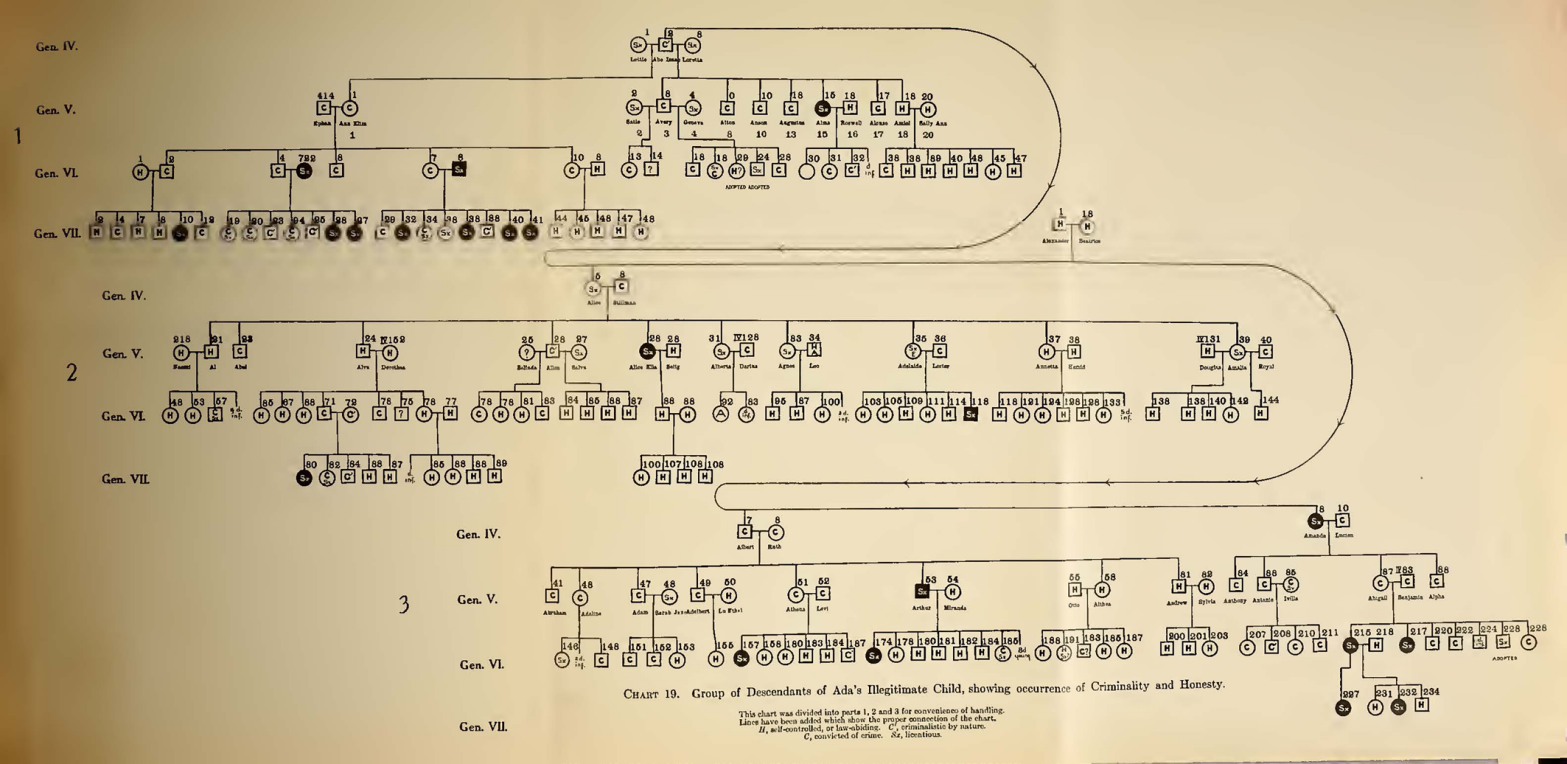
Occurrence of criminality in the Jukes (Estabrook 1916, p. 64)

For the eugenicist, that was evidence that became immediately apparent in pedigrees. By all means, the pedigree made it clear, according to Estabrook’s trained eye, that there was no good reason for regarding criminality as a “unit character” (Fig. 9).

As a result, since Estabrook deemed obvious that all feeble-minded Jukes were criminal and that “all the Juke criminals that I have known I regard as mentally defective” (Estabrook 1916, p. 67), he reckoned that, as advocated by Goddard, “the eradication of crime in defective stocks depends upon the elimination of mental deficiency” (Estabrook 1916, p. 85).

## Picture for Proof of Heredity and Environment

**Fig. 10:**
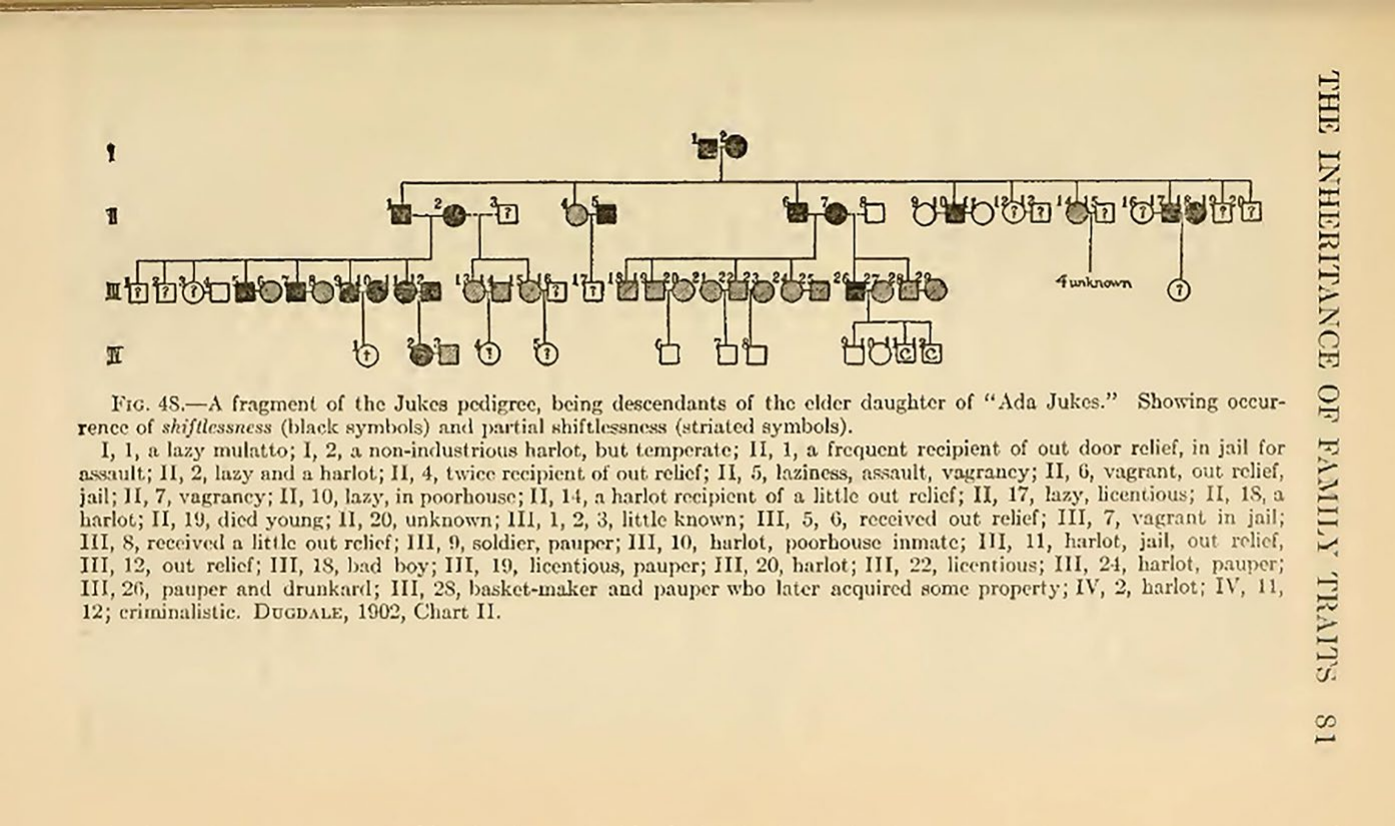
Pedigree showing shiftlessness in the Jukes (Davenport 1911, p. 81)

Before Dugdale’s Jukes record was found and the redoing of the Jukes case could be envisaged, Davenport made an early attempt of bringing the famous case on the side of the eugenicists in his important textbook *Heredity in Relation to Eugenics* (Davenport 1911). He tried to build a fragment of the Jukes pedigree showing the occurrence of “shiftlessness” as part of a strategy that attempted to demonstrate how complex traits such as “pauperism” were inherited as “unit characters” (Fig. 10).

**Fig. 11:**
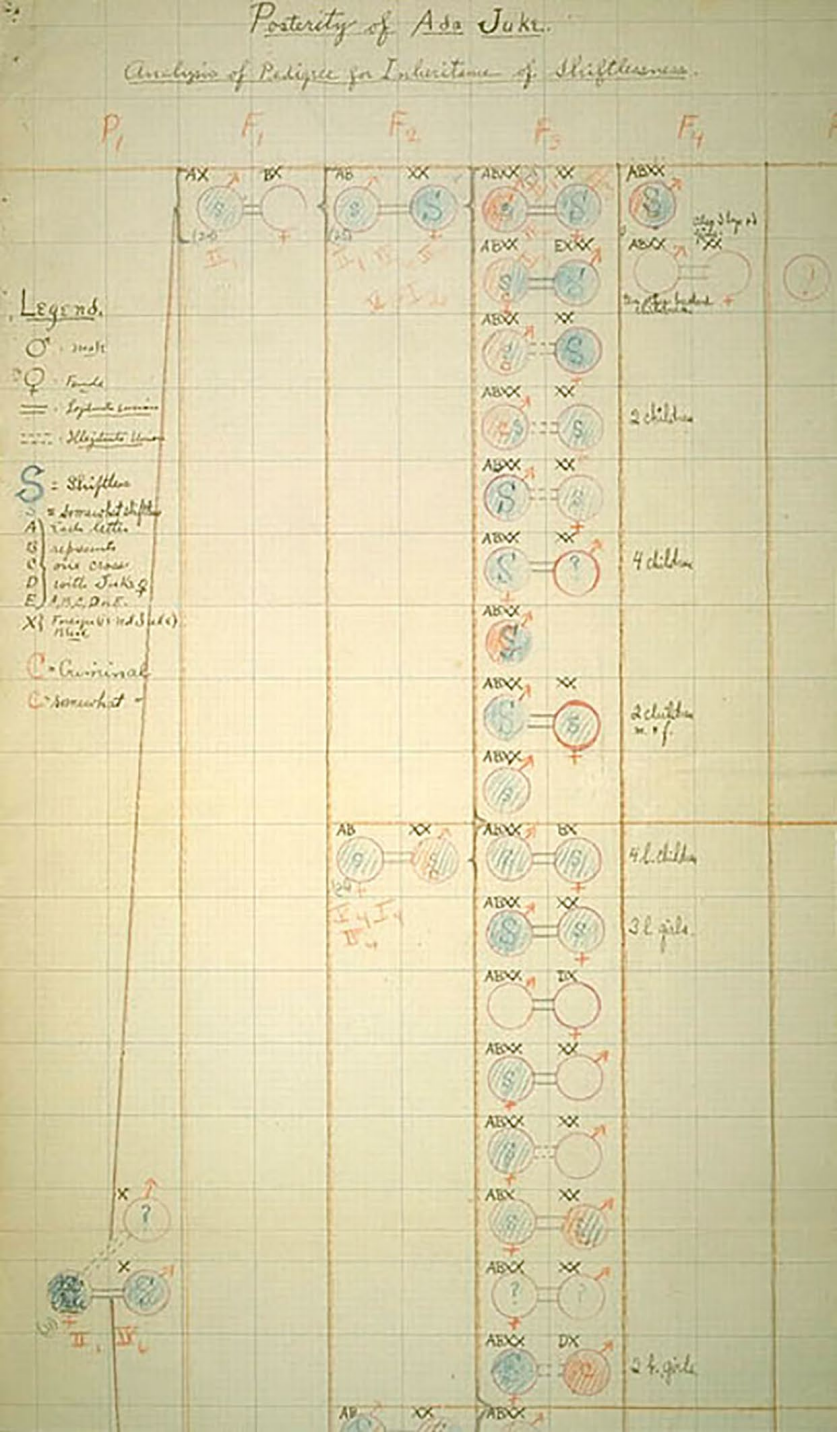
Posterity of Ada Juke - Analysis of pedigree of inheritance of shiftlessness. (Davenport, Charles B. Undated. “Posterity of Ada Juke: Analysis of pedigree of inheritance of shiftlessness.” American Philosophical Society, ERO, MSC77, SerI, Box 44, A:35–8.)

In his opinion, pauperism was the result of a complex set of causes and therefore only large family studies, such as those concerning the Jukes, were capable of proving that “barring a few exceptional conditions, poverty means relative inefficiency and this in turn means mental inferiority” (Davenport 1911, p. 80). The problem was that, up to that point, the Jukes had been directly studied only by Dugdale. As a consequence, Davenport resolved to build his pedigree on the sole basis of Dugdale’s text. Fig. 11 shows his attempt of translating Dugdale complex judgment of the various members of the family into Mendelian “unit characters.”

In this draft Davenport kept the tabular form used by Dugdale in his genealogical charts. Davenport then transposed the members of the chart into Mendelian symbols by “translating” the features that Dugdale summed up as short strings of text into two sets of opposed characters with incomplete dominance (with an intermediate state): shiftless / non shiftless (intermediate state: somewhat shiftless) and criminal /non criminal (intermediate state: somewhat criminal). Here, Davenport kept the letter notation system allowing to determine the crosses among the different “stocks” of the family and the notation system allowing to find the various individuals on Dugdale’s charts. These last two features disappeared in the published pedigree, which of course has also lost its original tabular arrangement. In the text, Davenport gives no explanation on how the data were gathered and how the Mendelian traits were determined. However, it was not difficult for the reader to guess that he derived traits from Dugdale’s work, which could expose Davenport to some serious criticisms (some of which will be discussed in the next section). In fact, not only did Davenport determine the heredity of “shiftlessness” on the base of characters extrapolated from a simplification of already simplified descriptions given in a book, but Dugdale himself had had to rely on the opinion of distant relatives or documents whose credibility could be easily called into question.

To avoid potentially embarrassing situations of this kind, when teaching at the ERO training programs Davenport often reminded students of the importance of investigating the conduct of individuals in their “natural habitat.” As a student reported in his notebook, “[we] cannot study habits of lions by their actions in Central Park.”^19^ Indeed, as historian of science Amir Teicher wrote, pedigrees had to stand inspection in a research area afflicted by the infeasibility of experimentation, and scholars interested in human heredity solved the problem by studying the mating conducted “inadvertently by humans throughout history” (Teicher 2020, p. 8). In this respect, “family histories […] functioned as ‘the protocol of an experiment that man unconsciously performed throughout generations’” (Teicher 2020, p. 8). However, in order to create pedigrees that could be confidently presented as sound research, it was necessary to prove that the information on which they were built was the result of first-hand investigation of the members of the family and not the collection of gossip from people more or less related with the studied individuals.

**Fig. 12:**
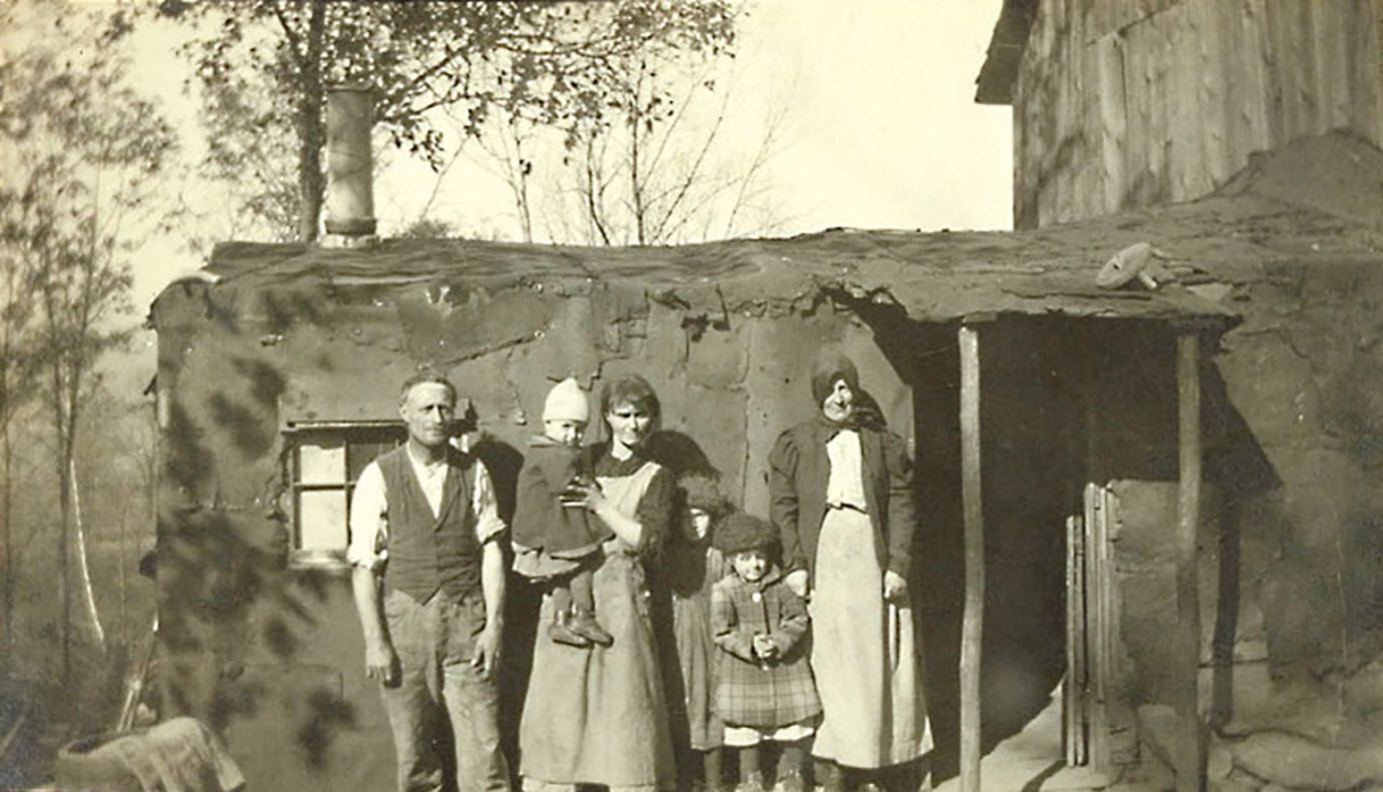
Field work for *The Jukes in 1915* (Estabrook, Arthur H. 1910 circa. “Field work for the Jukes in 1915, Arthur Estabrook photographs from Ulster County, New York.” University of Albany, SUNY, SPE, XMS 80.9, Box 1, Folder 1–27, ID# 1268.)

Sure enough, it was not uncommon for Davenport having to defend the ERO by claiming that “we have falsified no records and repressed no facts [as] is abundantly proven by our published work” (Davenport 1914, p. 9). He also pointed out that the ERO always endeavored to reduce the amount of error from unreliable sources by personally investigating the patients or interviewing as many as possible of their nearest relatives. To prove this, Estabrook integrated the data upon which his pedigrees were based with a number of photographs portraying the patients and the places in which they lived (Fig. 12). These photographs did not find a place in his published book, but they played an important role in exhibitions and other public events at which they were displayed. Much as footnotes are supposed to prove the scholarship of historical work, Estabrook’s images were meant to be the visual proof that the author actually tracked down the hundreds of Juke members who had spread out all over the United States in the last two generations. Building his diagrams on an organized collection of pieces of information (what we could call the Jukes archive) that could be connected with a visual reference was a way of supporting the professionalism of the investigation. This strategy provided a form of protection from allegations of biases in both the collection of data and the choice of the information used to build pedigrees.

Furthermore, it was particularly important for Estabrook to visually show that the Jukes were constantly reproducing their “faulty environmental conditions,” which was ascribed to their “germ plasm” (Estabrook 1916, p. 85). In fact, the photos with which Estabrook associated his pedigrees were part of an understanding of the environment as a hereditary factor itself. As Davenport put it in the conclusion of the preface he wrote for *The Jukes in 1915*, the work intended to show that “there is, indeed, no conflict between environment and heredity” (Estabrook 1916, p. iv). Indeed, Davenport maintained that “the controversy between heredity vs. environment has no good basis and it is fallacious to emphasize the distinction” (Davenport 1910, p. 21). Tracking down the two generations that eventually left the “cradle of the Jukes” was a way to show that the characteristic of the family remained mainly the same even outside their “ancestral spot.” Yet this was not framed as a denial of the role of the environment; on the contrary, because of their inherited characteristics, the members of the family inevitably tended to reproduce conditions of life that were identical with those they came from. Thus, the inherited conditions that they were bringing with them changed the environment where they were moving to. Here lies the importance of showing the Jukes within their own context, that is, their conditions of life and the miserable huts in which they were living. In this way the eugenics supporters could claim that the contraposition heredity/environment was an artificial one because, as a matter of fact, the environment itself was a product of heredity. Being able to incorporate the adversary’s position, and thereby denying the existence of any opposition at all, was the ultimate demonstration that one side of the debate had actually won. Estabrook, who ended his book by advocating either the confinement or the sterilization of people possessing “defective germ-plasm” —blatantly the most veracious actualization of pure hereditarian stances—presented his pedigrees as tools overcoming an artificial line of separation between the two “factors,” thus suggesting that, in order to overcome that dichotomy, it was de facto necessary to embrace the hereditarian cause.

## Standardizing Pedigrees and Answering Criticisms

**Fig. 13:**
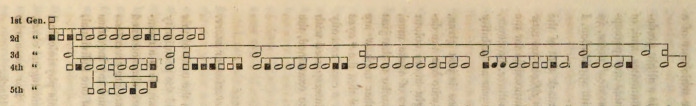
Pedigree showing the Inheritance of colour-blindness (Earle 1845, p. 350)

Being able to boast a specific “pedigree-facts” method—associated with the visual identity that this implied—would have helped boosting the ERO’s claims over being the institution par excellence for the science of eugenics. In order to do that, however, it was also necessary to develop a recognizable standard that could have been identified with that institution. At the time of Estabrook’s work, genetic pedigrees had already been used for some time, but a standard defining how they should be drawn did not exist yet, and the ERO aimed at filling that void (Resta 1993; Porter 2018). One of the main issues concerned the symbols that had to be used to represent males and females. As pointed out by the geneticist Robert G. Resta, this apparently trivial point became matter of contention because it reified divisions going along the lines of national preferences and, more importantly, it often became perceived as a marker of disciplinary affiliation—related to the well-known struggle between Mendelians and biometricians (Resta 1993). Some variants of the symbols classically related with Venus (♀) and Mars (♂) have been in use for centuries.^20^ However, though widely used in various kinds of pedigrees all over the 19th century, at the beginning of the 20th century these symbols were soon perceived as belonging to a specific British style—at least within the anglophone genetics community. The reason for this is to be found in the fact that the ERO, as the representative of American eugenics, championed the adoption of the circles for depicting females and squares for representing males. According to Edward Nettleship, an ophthalmic surgeon who was active when the ERO attempted to define a standard and uniform way of drawing pedigrees, Pliny Earle’s pedigree describing the inheritance of color blindness of his family in 1845 was credited as the first instance of the use of this convention (Anonymous 1913, p. 66) (Fig. 13).

Nettleship, who did not “approve of the use of squares and circles to indicate male and female,” also claimed that the origin of these modern symbols was probably very contingent: “Earle's diagram suggests that at the time he wrote he was unable to get any printer's symbols capable of use for his purpose except those employed in printing music” (Anonymous 1913, pp. 66–67).

These figures seem to be taken up by the ERO on the basis of their being more easily printed and the fact that they created less confusion on the page when there were many subjects on the same diagram (in particular, squares and circles have the advantage of taking less space) (Schott 2005). However, the ERO also attempted to fashion the pedigree tool in a way that could be identified with the institution promoting it and this resulted in a vague opposition between modern symbols (squares and circles, generally used by the Americans) and classical symbols (Venus and Mars, generally used by the British). Concerning the British counterpart, Resta wrote of a “Galton-Pearson style,” underlining the fact that the classical symbols were especially promoted by the biometricians (even though they were commonly used also by Bateson, Punnett and other British Mendelians) (Resta 1993, pp. 242–245). A paradoxical consequence of the antagonistic but inconsistent use of these two sets of figures can be seen in the context of the persistent problems between the (British) Eugenics Education Society and the biometrician Karl Pearson, one of the most prominent proponents of eugenics in the UK as well as Galton’s acolyte and biographer. When the conflict started to become more than simple disagreement, the Society retaliated against Pearson by recommending the use of the American Style despite its use of the Galton-Pearson style up to that point (Resta 1993; Mazumdar 1992).

The political and institutional relevance attached to aspects such as symbol choice lies in the fact that they can be seen as attempts to create a specific method in “genealogical-chart making” within the wider movement called “visual instruction movement.”^21^ Sometimes described as part of the field of visual education, which commenced at the beginning of the 20th century in the US, the movement involved educators keen to develop the use of visual tools in schools and universities. This could range from arranging educational exhibits to the establishment of slide and film libraries. Between the end of the 1910s and the beginning of the 1920s some American Universities began giving courses oriented towards the professionalization of “visual instruction.” One of the most important figures in this process was J. Harold Williams, an American psychologist, university educator and professor, who gave the first course in “graphic methods” at Stanford University. In 1924, Williams authored one of the most used textbooks of the movement, *Graphic Methods in Education*, which has an entire chapter devoted to graphic representation of “genealogical charts” (Williams 1924). Here, the proposed method was “the standard system adopted by the Eugenics Record Office” (Williams 1924, p. 248). In the exercises section, after having given some exercises requiring to copy a pedigree from Punnett’s *Mendelism*, it required highlighting the “English type of the characters” —that is, classical symbols of Venus and Mars (Williams 1924, pp. 248 and 253).

Williams, who later would become provost of Santa Barbara College (today University of California, Santa Barbara), carried out a doctoral study whose results were summarized in 1914 under the title *Relation of Delinquency and Criminality to Mental Deficiency* and in which family studies such as Dugdale’s *Jukes* and Goddard’s *Kallikak* are a primary reference.^22^ He was also a strong supporter of the Binet-Simon Measuring Scale of Intelligence Test in the United States and vehemently argued that the test was largely independent of verbal abilities and language acquisition. This belief matched with his engagement with developing visual tools in education, statistics and social sciences, and this engagement matched in turn with him having founded and directed the California Bureau of Juvenile Research, which was designated by Davenport as “official Western Representative of the Eugenics Record Office” (Stern 2005, p. 94). It thus seems consequential that the standard that he promoted for pedigree-making was the same promoted by the ERO.

The revaluation of the Jukes by the means of “pedigree-facts” was part of another related point that was important for the ERO to stress. Eugenics had to be built on such “facts,” but these were far from being enough. Pedigrees were meant to illustrate a family situation at a glance, but according to Laughlin they were only one of the three prerequisites that the practitioner of eugenics needed. The other two were “the knowledge of rules governing the inheritance of the traits in question” and, even more importantly, “the scientific skill with which the two foregoing factors are considered in connection with each other” (Laughlin 1922, p. 362). These considerations were particularly important because the ERO was obviously not the only institution working with pedigrees and because they allowed its members to exercise a certain dose of flexibility when answering to criticisms. This proved to be particularly useful when Davenport was forced to answer to the first virulent attack that was launched against the ERO modus operandi, which Lombardo called “one of the most public battles over the value of the new eugenic theories” (Lombardo 2008, p. 44). The attack came from the biometrical school of English eugenics. In 1913 and 1914, the Galton Laboratory published a detailed and scathing three-part paper that challenged the very foundations of the ERO pedigree method. The paper’s authors were Pearson, David Heron and Gustav Axel Jaederholm, who penned a criticism that assailed not only the quality of American Eugenics research, but the very reliability and professionalism of its work. In particular, Heron went so far as to claim that “the authors have in our opinion done a disservice to knowledge, struck a blow at careful Mendelian research, and committed a serious offence against the infant science of eugenics” (Heron 1913, p. 61). He did not shy away from claiming that the ERO field workers were instructed beforehand with the conclusions they had to reach, and the pedigrees were then determined accordingly, “whatever evidence against it” the research could actually show. Furthermore, Heron denounced the fact that the ERO instructed the field workers to make “a special search for the person or persons who are considered necessary for the support of the Mendelian theory” (Heron 1913, pp. 7 and 13). As the geneticist Leslie Clarence Dunn remarked, it was enough to bring the whole movement into disrepute (Dunn 1962).

Davenport took charge of a swift and equally vigorous response. Besides a detailed rebuttal in one of the ERO official Bulletins (Davenport 1914; Weeks 1914; Rosanoff 1914), in order to emphasize the public and “national” dimension of the issue he placed a defence in the columns of the *New York Times* (with an article tellingly entitled “American Work Strongly Defended — English Attack on Our Eugenics”) and *Science* (Davenport 1913a, 1913b). He focused on the argument according to which the criticisms were based on the ignorance of the pedigree-method as it was used in the ERO. He maintained that the pedigrees are “simply non-committal” because, to be meaningful, they had to be read together with the detailed descriptions of the family members given in the text and it was the long experience of the practitioner, ensured by his trained eye, that could make sense of the complex of pedigree-facts, the Mendelian theory and their mutual and not always clear connections. Consequently, pedigrees could give insights of a case “at a glance,” but only to those with the right training and experience to read them and to evaluate them in connection with the contingences of the case. In other words, only those possessing the right “analytical tools” could actually understand the meaning of what was at hand, and the right “analytical tools” could be learned only under the supervision of the researchers working at the ERO.

By the same token, a work of that kind was important because it gave the opportunity of showing the capacity of the ERO pedigrees method to delve beyond those typically used by genealogists. As Laughlin summarized, the “usual outline of the genealogist […] is merely the skeleton.”^23^ As remarked by Wilson, Laughlin repeatedly stressed that more classical genealogies, used by researchers as Dugdale, lacked the “detailed description of the natural, physical, mental, and temperamental qualities of each member listed” —even though the claim defies any reading of Dugdale’s text.^24^ Only after having obtained those kinds of information from a qualified and reliable field worker who knew what to look for, was it possible to obtain a “record of practical pedigree-value, one which can be used in tracing the descent and recombination of natural qualities within the family-tree.”^25^ The success of this operation can be judged by the use to which the Jukes’ case was put into educational and pedagogical practices. By way of example, in the 1920s–1930s the science high school teacher and pedagogical researcher Amer M. Ballew recommended for biology classes a unit of “practice in construction and interpretation of hereditary diagrams” (Ballew 1929, p. 355), in which students were learning the craft of tracing human ancestry back to generations on the bases of instances such as the coat color of guinea pigs and the interpretation of pedigrees of the Juke family.

## The Triangle of Life and Professionalization

ERO’s projects such as Estabrook’s were geared to promote the exclusive authority of one preeminent method of work, the one based on the so-called pedigree-facts as specifically fashioned by the ERO.^26^ So, for example, in 1917, Estabrook could use the impact force of his high-test method to claim boldly the meaninglessness of Sam Juke’s case. Sam was a young Juke who was initially being adopted by a “kind-hearted widow” and afterwards by a family where “firm discipline was joined to great kindness” (Estabrook 1917, p. 41). His story was popularized by the Children’s Aid Society of New York to show that in those contexts he could achieve “a thoroughly good reputation in the community, and [could strike] out for himself with a good moral foundation on which to build his subsequent career” (Estabrook 1917, p. 41). Estabrook felt entitled to rebut that he knew well the case in question, and it was one for which no pedigree-facts could be established because it was not possible to know who the father was. As a result, in Estabrook’s eyes Sam became, contrary to the Children’s Aid Society intentions, a demonstration of the fact that “the assumption that the environment can counteract heredity cannot be proved by any example of a production of good traits in a changed environment in any individual when the traits which that individual inherits are not known” (Estabrook 1917, p. 42). This modus operandi had the great advantage of denying the legitimacy and seriousness of the investigations carried out by one of the two sides of the controversy. It aimed at persuading the scientific community that a dichotomy of hereditarian and environmental factors were only a bad way of framing a problem that did not exist, while at the same time asserting the clear superiority of a way of conducting research elevated as the only sound method of research.

**Fig. 14:**
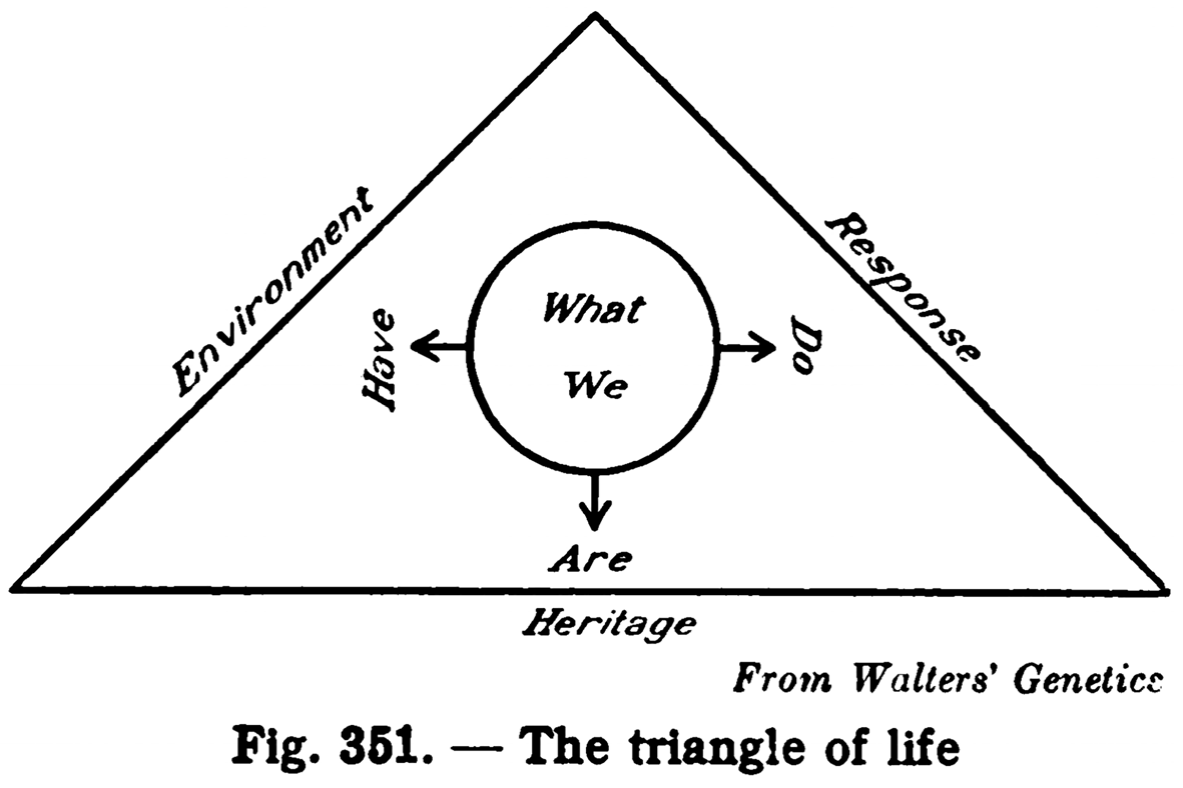
The triangle of life in an American biology high-school textbook (Peabody and Hunt 1924, p. 541)

This strategy would later have its own diagrams in the form of the famous “triangle of life,” generally depicted as an equilateral triangle whose sides represent the three “fundamental factors in the development of the family and race.”^27^ This diagrammatic representation became a staple of eugenics pedagogical approach and was to be found in several biology high-school and university textbooks (Fig. 14),^28^ as well as in posters to be used in settings such as expositions and public fairs.^29^

Here, the position held by the opponents of eugenics benefited from the doubling of its components (training was framed as something different from environment and was considered as a “factor” in its own right), but the diagram allows the depiction of the remaining side, “heredity,” as the base upon which the rest depends, thereby conferring it a role that the other two aspects cannot claim to have: “while training and environment are essential to the formation of character and physical development, heredity will ever remain the determining factor of the three and be regarded as the base of the triangle” (Mous 1913, p. 227).

**Fig. 15:**
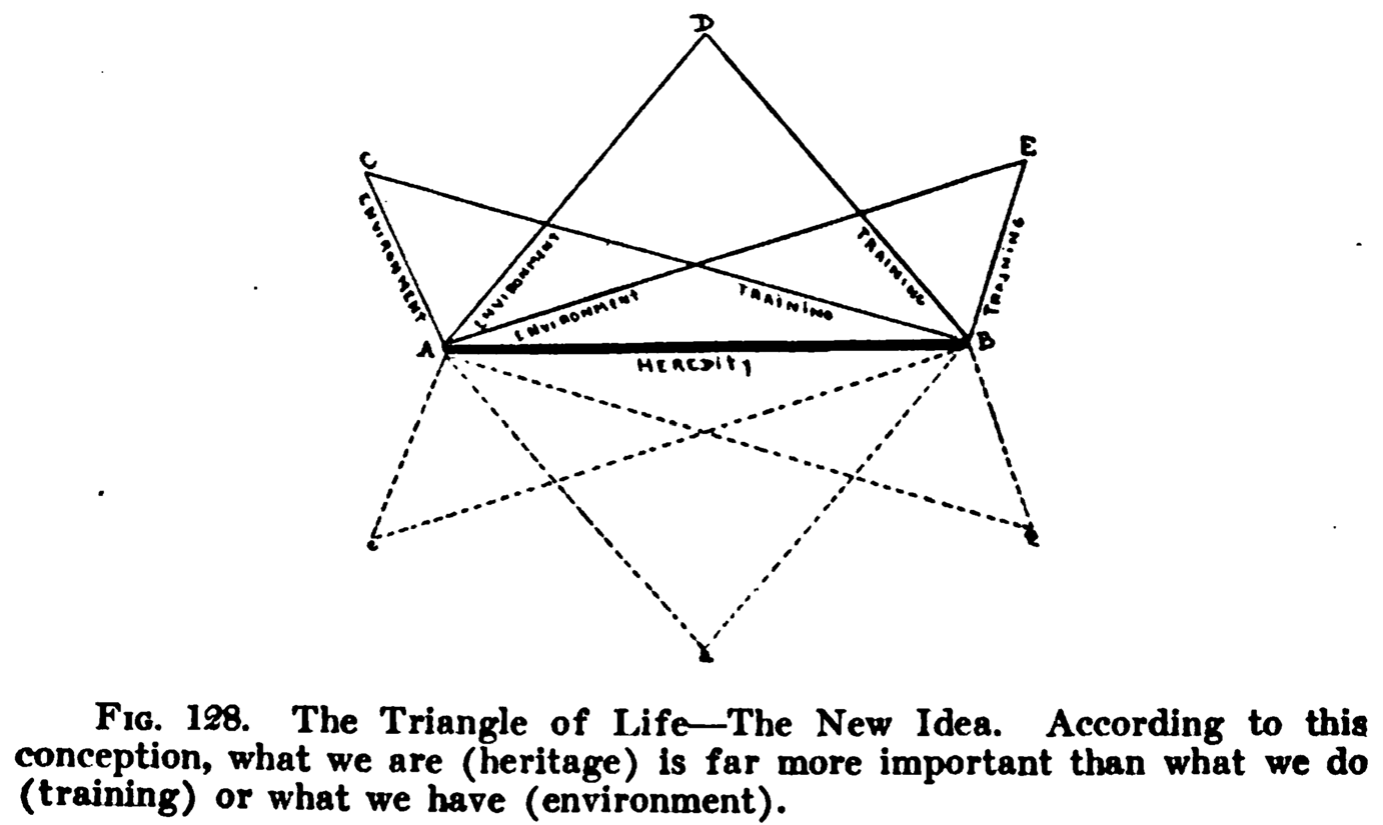
Triangle of life with heredity as “substantial basis” and environment and training as “swinging factors” (Goldsmith 1922, p. 379)

A more elaborate representation of the concept depicted heredity as the “substantial base” around which the other two “subordinate factors” would swing according to the specific circumstances of the family (Fig. 15).

**Fig. 16:**
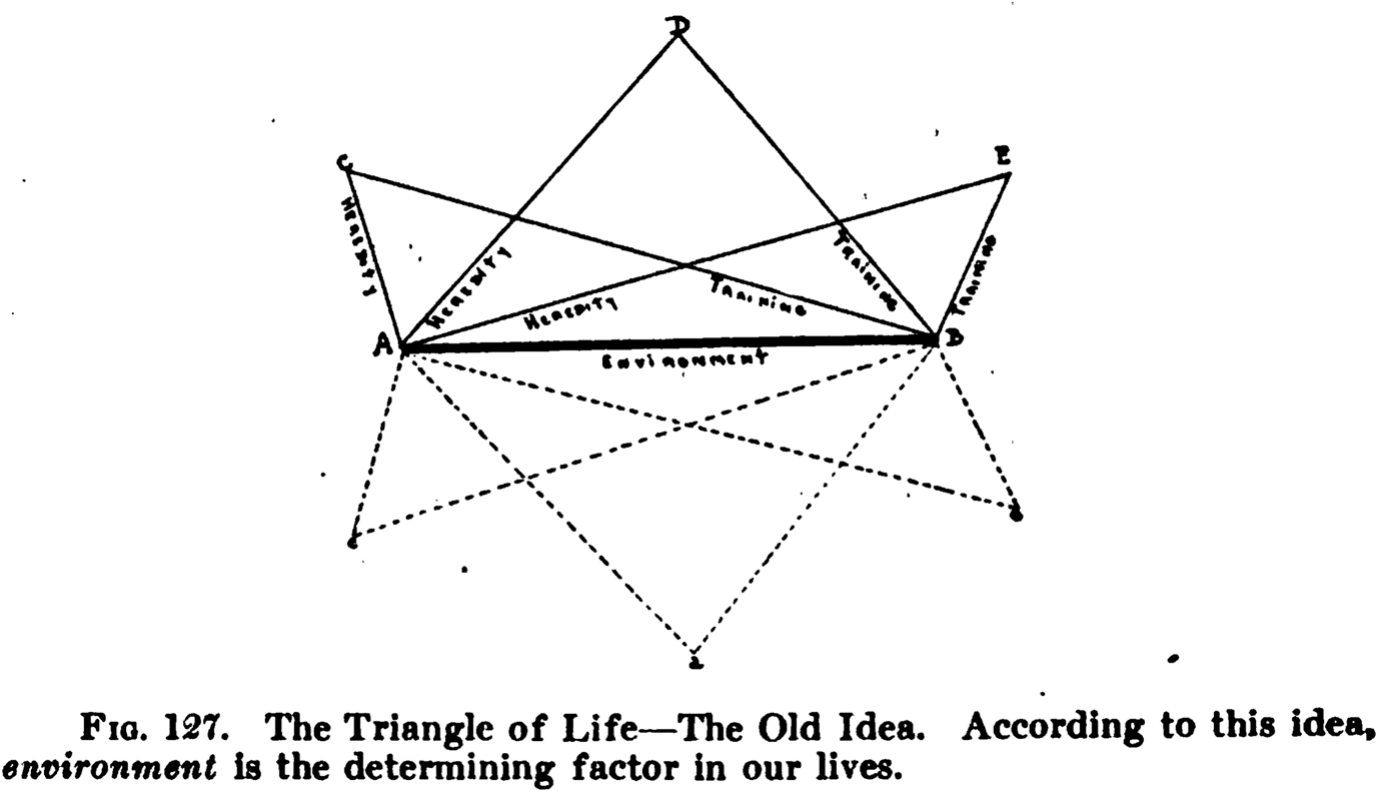
Triangle of life, the old idea (Goldsmith 1922, p. 378)

In this perspective, “old reformists” such as Dugdale differed from the new ones (the eugenicists) for deeming the environment as the substantial basis and heredity and training as the two subordinate, swinging factors (Fig. 16).

The positive outcome of this strategy allowed Laughlin to advance the already mentioned definition of “pedigree-facts” in his important *Eugenical Sterilization in the United States*, which contained the “Model Eugenical Sterilization Law,” the groundwork on which several US states modelled their laws for forced sterilization for “socially inadequate” people. Here, “pedigree-facts” are “authentic records which describe in detail the specific traits under consideration, and the distribution of these traits among the several members of the family-tree” (Laughlin 1922, p. 362). Indeed, as soon as the need of trying to answer the question of the relative parts played by heredity and environment in the emergence of pauperism and criminality was pushed aside because it was deemed a sorted-out issue, pedigree construction could be marketed as the only framework par excellence capable of establishing the basic elements upon which any level of policy had to be made. Works such as those produced by Estabrook had a three-fold purpose in this process: establishing the primacy of heredity in determining social problems, establishing the primacy of the pedigree method in the analysis of heredity, and establishing the primacy of the Eugenics Record Office in the use of the pedigree-method.

Estabrook was given voice regarding the constitutionality of forced sterilization practices in the US on the ground that he was the author of *The Jukes in 1915*. As such, he could authoritatively call into question the choice of Warren Wallace Smith, a prisoner convicted for rape and incest, as a test case for the constitutionality of the Indiana Sterilization Act of 1907 (Laughlin 1922; Stern 2007). His assertion was based on the fact that the judges could not have the expertise deemed necessary to ground their choice because they did not know “the present day methods of eugenics” (Laughlin 1922, p. 256). Indeed, Estabrook stated that the pedigree-method was necessary in cases like this in order to prevent patients from unwarranted sterilisations, which in turn implied that ERO trained practitioners were necessary to sort out the issue.

**Fig. 17:**
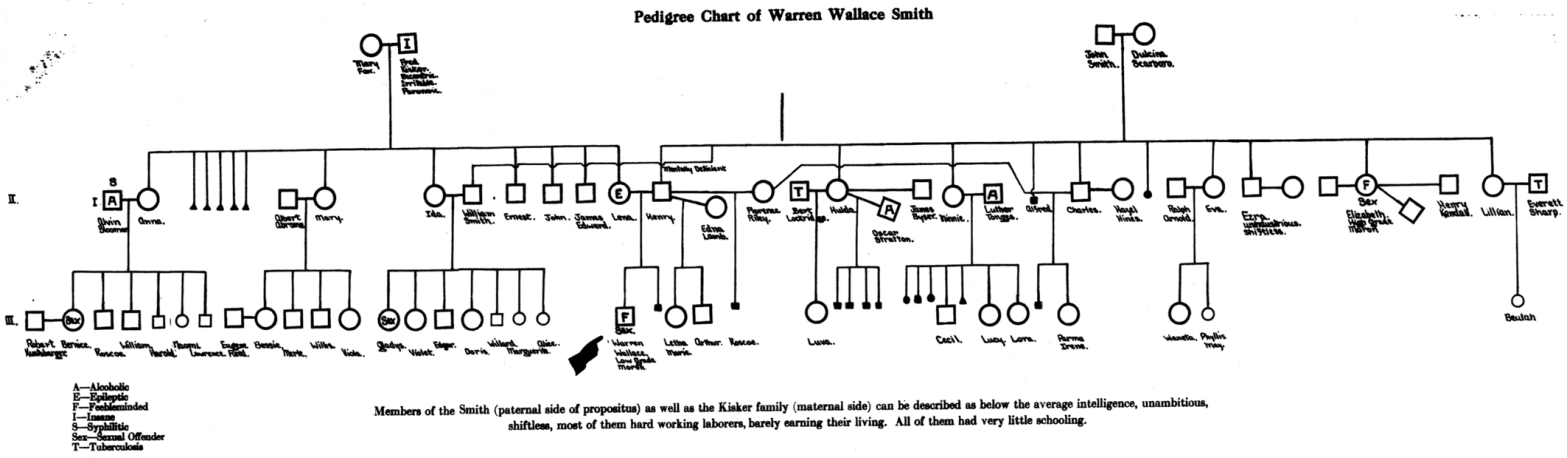
Pedigree chart of Wallen Wallace Smith (Laughlin 1922, p. 321)

In this way, ERO members could both exert pressure for having sterilization laws passed in many US states and proposing themselves as the keepers of the method that could protect people from a wrong diagnosis. Accordingly, Laughlin promoted the ERO as the center par excellence for providing the necessary expertise for such cases by displaying pedigrees produced by ERO members and promoting them as the demonstration of the analytical power of the method around which the institution was trying to build his disciplinary identity. As a consequence, Estabrook was asked to carry out a complete analysis of Smith. The outcome of the task was Smith’s “short pedigree,” which was proposed as “typical of the scientific studies that should be made, and required by law as a matter of routine in connection with every case proposed for eugenical sterilization” (Laughlin 1922, p. 318) (Fig. 17).

Another, powerful move was the campaign for the need of professional and certified eugenics field workers. Laughlin deemed that there was the need of experts in the pedigree-method entitled to certify that the analysis of the families was performed under due course of professional practice. Thus, there was the need for formalizing the position of “professional eugenicist […] who should be aided by an ample corps of assistants” (Laughlin 1922, p. 102). That need had been met in the previous decade in the United States by the ERO, which had been training eugenicist field workers in intensive summer courses specifically devised to instruct aspiring eugenics caseworkers. As I have already mentioned, Estabrook was the product of the first of those courses and Laughlin could boast of having already 150 investigators like him ready for work.

The culmination of this plan was meant to be the creation of an “Office of State Eugenicist,” which in turn required an organ establishing legal qualifications for public office, that is to say, ensuring the official “Qualification of State Eugenicist.” It was of course implied that there was only one institution in the US that could meet the requirements here outlined, and that was the ERO. Laughlin was thereby establishing the fundamental need of the position of the “trained eugenicist,” who “should be well paid, and should be required to devote his entire time and attention to the duties of his office” (Laughlin 1922, p. 443). In his words, “whenever a degenerate line is located, the State Eugenicist must then use persons trained in modern pedigree-studies to make further analysis of the facts” (Laughlin 1922, p. 457). Exemplary cases such as the new version of the *Jukes* and the skills of the field worker who conducted it provided, in Laughlin’s opinion, the best proof of the value of such approach.

Laughlin’s view scored a significant victory when Estabrook was hired to testify in the ground-breaking Supreme Court case *Buck vs. Bell*, the trial that set a legal precedent according to which the public authority could sterilize patients deemed afflicted by “hereditary defects” without violating the due process required by the 14th Amendment to the US Constitution. After a cursory examination of Carrie Buck (the girl plaintiff that had to be sterilized because she was defined a “moral degenerate” after being raped by a nephew of her foster parents) and her mother, Estabrook claimed that his expertise in pedigree studies made him conclude that both women were plausibly part of a “defective strain.” More to the point, Estabrook defined the Buck family as a contemporary analogue of the Jukes. Indeed, the argument that he developed revolved around the equivalence of Carrie Buck’s family to “the original Jukes” in “its propensity to crime, poverty, and feeblemindedness” and, as Lombardo stressed, “Estabrook’s testimony was a key piece of evidence used to support the court’s conclusion declaring the legal validity of [the] sterilization law” (Lombardo 2012, p. 224). A work such as Estabrook’s re-evaluation of the Jukes was so influential because it showed the extent to which the modern ERO pedigree method, combined with the knowledge and the judgment provided by eugenicists trained by that institution, could change the interpretation of one set of data—in fact, beside the two last generations, the data used by Estabrook were the same provided by Dugdale. Using the “new” Jukes as the touchstone for such a national discussion as *Buck vs. Bell* represented the ultimate certification of the supremacy of the ERO’s approach to his own field of study and the definite disqualification of the others.

## Conclusions

Estabrook’s redoing of the Juke family study on behalf of the Eugenics Record Office was presented as an extension of Dugdale’s original investigation. However, Estabrook’s work also involved the replacement of Dugdale’s eclectic method, encapsulated in his genealogical charts, with the analysis of “pedigree facts” as it was advocated by the ERO. The powerful synoptic impact of this kind of graphic representation was instrumental in accomplishing the redefinition of the Jukes as an icon of strictly hereditarian approaches. In particular, even though the role of the environment was always recognized, these diagrammatic tools fostered an intrinsic hereditary understanding of the transmission of social and psychological “unit characters.” As a result, the ill-defined and difficult-to-discriminate aspects under examination—such as inclination for crime—were predominantly traced back to the inheritance of feeble-mindedness, thereby making the Jukes a staple model of eugenic pedigree studies. Estabrook’s work was also part of a series of projects geared to promote the exclusive authority of one preeminent method of work over other possible approaches to a young discipline—eugenics—and to promote the institution associated with that method—the ERO—over rival actors. The iconic nature and the persuasive power of such an emblematic case was instrumental in asserting the superiority of the ERO’s “analytical tools” and, by extension, the necessity of extending the ERO’s authority, up to the point of requesting the institutionalization of an official “Qualification of State Eugenicist” controlled by that institution.

## Footnotes

1 To mention one of many possible cases, the butterfly *Papilio dardanus*—the African mocker swallowtail—has been studied as one of the main textbook examples of ecological genetics since the latter has been defined as a full-fledged discipline. See Ford (1936) as the first comprehensive account of the case.

2 In early genetics, the term “unit character” designated an observable trait passed down in generations as a single entity—that is, a unit of inheritance which is controlled by a “unit factor.” See Schwartz (2002).

3 For more on ERO trained caseworkers, see Bix (1997); for more on the ERO in general, see Allen (1986).

4 The “presence and absence” hypothesis in genetics postulated that what is described as a Mendelian “pair of characters” is actually due to the presence and absence of a single character. See Shull (1909).

5 Anonymous (1911). “Student exercise on Jukes family,” American Philosophical Society, ERO, MSC77, SerI, Box 44, A: 35–7.

6 Shepard (1884, p. 4), quoted in Lombardo (2012, p. 211).

7 Even though Francis Galton laid some of the theoretical foundations for a science of the “betterment of the human race” in his 1869 book *Hereditary Genius: An Inquiry into its Laws and Consequences*, the very term “eugenics” did not exist until 1883 when he coined it in his *Inquiries into Human Faculty and its Development*. Dugdale died shortly after that, the 23rd of July 1883.

8 Estabrook, Arthur H. 1914. “The Juke data, 1914 August 1.” New York State Modern Political Archive Collections, Arthur H. Estabrook Papers, Notes, 1908-1925, Box 2 (5-notes), Folder 3.

9 Anonymous. 1912. “‘Degenerate family cost state heavily,’ about rediscovery of Dugdale's charts of real names of Jukes family members (1/18/1912).” University of Albany, SUNY, Estabrook, SPE, XMS 80.9, Box 1, Folder 1–35.

10 At that time Estabrook was a young researcher who was trained in zoology and who had just completed his John Hopkins University doctorate. His training in the ERO summer program would mark the beginning of his reputation as an expert in eugenics.

11 Anonymous (1875a), quoted in Lombardo (2012, p. 209).

12 Anonymous (1880), quoted in Lombardo (2012, p. 210).

13 See for example Anonymous (1874a, b, 1875b, c, f).

14 The Prison Association of New York (today the Correctional Association of New York) was founded in 1844 to improve the conditions of prisoners while in jail and to provide them with assistance when released.

15 Shepard (1884, p. 4), quoted in Lombardo (2012, p. 211).

16 See Anonymous (1875d, e).

17 Davenport, Charles B. 1911. “Charles Davenport to Arthur H. Estabrook” (8 January 1911). American Philosophical Society, Charles B. Davenport Papers, B D27, SerII, Folder: “Estabrook, Arthur H. #1”, quoted in Bender (2011, p. 192).

18 Laughlin was heard in his capacity as “expert eugenic agent,” a position that he held at the House Committee on Immigration and Naturalization from 1921 to 1931.

19 Anonymous. 1915. “Meeting of the field workers,” American Philosophical Society, ERO, MSC77, SerVII, Box 1: 5.

20 These symbols were derived from contractions in Greek script of the Greek names of these two planets. See Schott (2005).

21 For the institutional development of the “Visual Instruction Movement” in the US see, for example, Saettler (2004).

22 Williams, Harold J. 1914. “Relation of delinquency and criminality to mental deficiency.” In Williams, Harold J. and Lewis M. Terman. Report of the Psychological Survey of the Whittier State School, 30–50. Typewritten document. Stanford University.

23 Laughlin, Harry H. Undated. “A few points to observe in writing up notes.” Truman State University, Pickler Memorial Library, Harry Laughlin Papers, Eugenics Field Worker-Material Used in Classes-1921, Box C-2-4, Folder 9, quoted in Wilson (2008, p. 168).

24 Woods (1920, p. 77), quoted in Wilson (2008, p. 168).

25 Woods (1920, p. 77), quoted in Wilson (2008, p. 168).

26 Another notable example of the convenience and authority conferred by the use of diagrams can be found in Norton Wise’s study of the growth of curves in theoretical and experimental physics in mid-19th century Germany. See Norton Wise (2018, pp. 169–174). I am grateful to Jean-Baptiste Grodwohl for pointing out to me the similarities of the two cases.

27 This definition has been printed several times in many different venues. See for instance Keister (1916, p. 48).

28 See Selden (2007). A notable example can be found in the university textbook *Genetics: An Introduction to the Study of Heredity* by Herbert E. Walter, where the explanation of the diagram of the triangle of life is used as the outright beginning of the book.

29 Anonymous (1929). “Unfit human traits” and “Triangle of life,” American Philosophical Society, Fitter Families Collection, 2000.1285.
